# ENHANCE (Tailored Intervention for Brain Health and Cognitive Enrichment for Cognitive Health), a Coach-Supported Digital Intervention for Dementia Prevention in Underserved Older Adults: Co-Design and Usability Study

**DOI:** 10.2196/91999

**Published:** 2026-07-29

**Authors:** Clare Tsz Kiu Yu, Gill Livingston, Louise Allan, Richard Boczko, Sue Boex, Youngjun Cho, Kealan Forristal, Rachael Hunter, James Jamison, Fahima Khatun, Carl Leckstein, Louise Marston, Vrushti Mehta, Naaheed Mukadam, Hee Kyung Park, Shi Qiu, Greta Rait, Penny Rapaport, Joanne Reeve, Longbing Ren, Anne GM Schilder, Aneesha Singh, Andrew Sommerlad, Alanah Spillane, Liz Steed, Michelle Xu, Kai Yao, Ruan-Ching Yu, Sergi G Costafreda

**Affiliations:** 1 Division of Psychiatry University College London London United Kingdom; 2 North London NHS Foundation Trust London United Kingdom; 3 Medical School University of Exeter Exeter United Kingdom; 4 Faculty of Health Sciences University of Hull Hull United Kingdom; 5 Department of Computer Science University College London London United Kingdom; 6 UCL Interaction Centre University College London London United Kingdom; 7 Social, Genetic & Developmental Psychiatry Centre King's College London London United Kingdom; 8 Centre for Applied Health Research University College London London United Kingdom; 9 UCL Ear Institute University College London London United Kingdom; 10 Division of Psychology and Language Sciences University College London London United Kingdom; 11 Department of Primary Care and Population Health University College London London United Kingdom; 12 Samsung Medical Center Seoul, Seoul Republic of Korea; 13 Department of Neurology and Neurological Rehabilitation School of Medicine, Shanghai Disabled Persons’ Federation Key Laboratory of Intelligent Rehabilitation Assistive Devices and Technologies, Shanghai Yangzhi Rehabilitation Hospital (Shanghai Sunshine Rehabilitation Center) Tongji University Shanghai China; 14 Wolfson Institute of Population Health Queen Mary University of London London United Kingdom; 15 Centre for Neuropsychiatric Genetics and Genomics Division of Psychological Medicine and Clinical Neurosciences Cardiff University Cardiff United Kingdom

**Keywords:** aging, app, behavioral health, cognitive training, coproduction, dementia, older adults, participatory research

## Abstract

**Background:**

Digital multidomain interventions hold promise for dementia risk reduction; however, populations at higher dementia risk, including those experiencing socioeconomic and educational disadvantage, remain underrepresented in trials, and engagement with digital interventions often declines over time. Coproduction and blended models that combine digital tools with human support may improve reach, acceptability, usability, and sustained engagement. Designing interventions that are usable and acceptable for individuals facing structural, educational, or digital barriers (underserved groups) is therefore likely to produce solutions that are both accessible and scalable for the wider midlife and older adult population.

**Objective:**

This study aims to describe the coproduction process used to develop ENHANCE (Tailored Intervention for Brain Health and Cognitive Enrichment)—a coach-supported digital intervention targeting 10 modifiable dementia risk factors in older adults from underserved groups—and report key outputs and lessons learned for equitable digital prevention design.

**Methods:**

We coproduced ENHANCE between July 2023 and February 2025 using a multistage development process guided by the Medical Research Council framework for complex interventions and the Double Diamond design model. The person-based approach informed user-centered guiding principles (key design objectives), while behavior change content was operationalized using behavioral change theories. Coproduction followed 4 phases. The Discovery phase explored barriers to engagement with existing digital materials and identified candidate components for each dementia risk-factor module. The Define phase translated these insights into guiding principles and blueprints of each risk-factor module integrated with behavioral change components. The Design phase involved iterative co-production and usability testing of prototypes. The Delivery phase evaluated a high-fidelity prototype through a 1-week usability study with coaching support. Contributors included 162 research participants recruited from underserved community settings, 33 patient and public involvement contributors, and 4 human–computer interaction experts. Throughout development, coproduction focused on reducing literacy, digital confidence, and cultural barriers to maximize usability across diverse adult populations.

**Results:**

Coproduction produced (1) evidence-informed module strategies for targeted dementia risk factors; (2) a set of guiding principles to ensure low-literacy, culturally relevant, and accessible content, supporting both equity of access and wider population usability; (3) a meadow-themed app integrating tailored check-ins, educational videos, cognitive training games, and in-app messaging; and (4) a structured coaching model, including onboarding, brief follow-up, and accompanying coaching manuals. Iterative testing and refinement improved navigation, simplified language, reduced text burden, and ensured the use of familiar and accessible game formats, resulting in a feasibility-ready prototype.

**Conclusions:**

ENHANCE is a coproduced, coach-supported digital intervention designed to be accessible for underserved midlife and older adults at increased dementia risk, with design features to support accessibility, engagement, and scalability across the wider aging population. The development process illustrates how integrating coproduction with behavioral science and usability methods can support principled intervention design for equitable digital dementia prevention.

## Introduction

Dementia is an urgent global health issue, with prevalence expected to almost triple—from 57 million in 2019 to about 153 million by 2050 [1], making prevention a priority. Nearly 45% of dementias could potentially be prevented by addressing 14 modifiable risk factors, including hypertension, diabetes, physical inactivity, and obesity [2], and multidomain interventions targeting several risk factors simultaneously have shown preventive benefits [3]. Large multidomain dementia prevention trials, including the US POINTER trial (N=2111) and Maintain Your Brain trial (N=6104) have demonstrated cognitive benefits of structured multidomain lifestyle intervention on healthy midlife and older adults [4-6].

However, dementia risk is disproportionately higher among socioeconomically disadvantaged groups and ethnic minority groups [7-10]; yet, these populations remain underrepresented in dementia-prevention trials [11,12]—a recent scoping review identified only 7 multidomain digital dementia prevention programs, largely evaluated among highly educated women [12], and ethnic minority participation has consistently fallen below population norms [10]. This highlights the need for dementia prevention programs that are inclusive of underserved, high-risk populations. In this study, “underserved” refers to midlife and older adults at elevated dementia risk who are underrepresented in prevention trials, including those from socioeconomically deprived areas, ethnic minority backgrounds, or with lower educational attainment.

Traditional dementia prevention trials have predominantly relied on face-to-face, center-based delivery in group or individual formats [13-15]. These approaches are resource-intensive—for example, the FINGER trial estimated intervention delivery costs of approximately US $640 per person in staff and session costs and requires significant staffing, venue provision, and participant travel. Ethnic minority groups and those from lower socioeconomic backgrounds remain underrepresented in such trials [10-12], and center-based formats may compound this: English-only delivery can exclude those with less English proficiency [16], while travel costs and time away from work create additional burdens for those from lower socioeconomic backgrounds [17,18]. Together, these limitations restrict the scalability and equity of traditional dementia prevention approaches.

Digital technologies offer a promising means of addressing these gaps. Remote delivery reduces geographical and scheduling barriers, broadening reach to underserved populations [19], while digital platforms lower costs associated with staffing, venues, and in-person sessions. Rising technology use among older adults supports the feasibility: by 2022-2023, 81% of people aged 65 years and older were using the internet at least monthly [20], and smartphone ownership among lower socioeconomic groups reached 67% in 2018 and has likely grown since COVID-19 [21]. However, sustaining long-term engagement remains a key challenge. In PRODEMOS, a 1-year multidomain dementia-prevention trial, app usage declined from 91% in the first 2 weeks to 41% by the third month [22], and in the LETHE trial (N=159), a 2-year app-based multidomain lifestyle intervention, only half of participants engaged daily, with mean session durations of just 42 seconds [23].

Several design limitations in existing interventions may underlie these challenges. Theoretical grounding has been inconsistent, with only 35% of app-based interventions explicitly referencing a theoretical framework [24]. Without theory-driven design targeting motivation and habit formation, interventions may fail to induce sustainable behavioral change. Human support has also been limited—in Active Brains, support comprised up to 3 brief telephone calls over 1 year [25], while PRODEMOS and HATICE relied primarily on online or in-app messaging with limited real-time interaction [22,26]. Insufficient human contact may reduce accountability and motivation over time. Existing tools have also been criticized for a lack of usability and accessibility, with platforms poorly suited to the varying digital literacy levels, age-related, and diverse needs of older adults [12]. These shortcomings may partly reflect limited user involvement during development. Review reported that only 2 out of 40 interventions included older adults in their development [27], and that reporting of key participant characteristics such as ethnicity and socioeconomic status in co-design activities remained scant [26]. Together, these gaps suggest that existing digital dementia prevention interventions have not been sufficiently designed with the older adults they aim to serve.

Coproduction offers a structured approach to addressing this gap. As a collaborative process in which researchers, developers, and users work as equal partners [28], coproduction facilitates the development of interventions that are relevant, acceptable, and usable beyond controlled research settings [29], enables early identification of user needs and potential design limitations [30-32], and fosters user trust in the final product [33]. Blended approaches combining digital tools with human support and behavior change techniques (BCTs) are associated with higher engagement and actual behavioral change over time [34-36].

In summary, existing digital dementia prevention interventions have predominantly been designed for and evaluated in populations with higher educational attainment and greater digital literacy, risking widening inequalities. Coproduction—particularly when purposively including older adults from underserved groups—can help ensure that intervention content, delivery, and support are better aligned with those at highest risk. Guided by these gaps, we developed ENHANCE (Tailored Intervention for Brain Health and Cognitive Enrichment), a coach-supported, app-based intervention targeting 10 modifiable dementia risk factors—physical inactivity, obesity, diabetes, hypertension, hearing loss, smoking, excessive alcohol use, social isolation, depression, and cognitive inactivity—delivered through 9 dedicated lifestyle modules and a suite of cognitive training games. ENHANCE was coproduced using the person-based approach [37] and behavioral change wheel [38], with particular emphasis on accessibility and relevance for underserved groups.

## Methods

The development of ENHANCE took place between July 2023 and February 2025.

### Ethical Considerations

This study complied with the Declaration of Helsinki and received ethical approval from the University College London Research Ethics Committee (reference number 24235/001) on 19 June 2023.

Before enrolment, research participants received a participant information sheet, had the study procedure explained, and were informed of their rights to withdraw at any time. Written informed consent was obtained from all participants.

Data were pseudoanonymized using an alphanumeric code. The code list and consent forms were stored separately from the anonymized data in locked cabinets (paper) and on a password-protected, encrypted institutional drive (electronic), accessible only to the core research team.

Participants received shopping vouchers for their time: £20 (GBP £1=US $1.27 as of Jan 1, 2024) for 45-minute usability or focus group sessions (in Discovery, Define, and Design phases) and GBP £80 in total for the 1-week Deliver phase testing (GBP £25 for onboarding, GBP £25 for postsession feedback, and GBP £30 for app use), in line with National Institute for Health and Care Research (NIHR) payment guidance [39].

PPI and HCI experts participated in an advisory capacity and were not classified as research participants; written consent was therefore not required. PPI received £20 per hour reimbursement per NIHR recommendation [39].

### Development Frameworks

**Figure 1:**
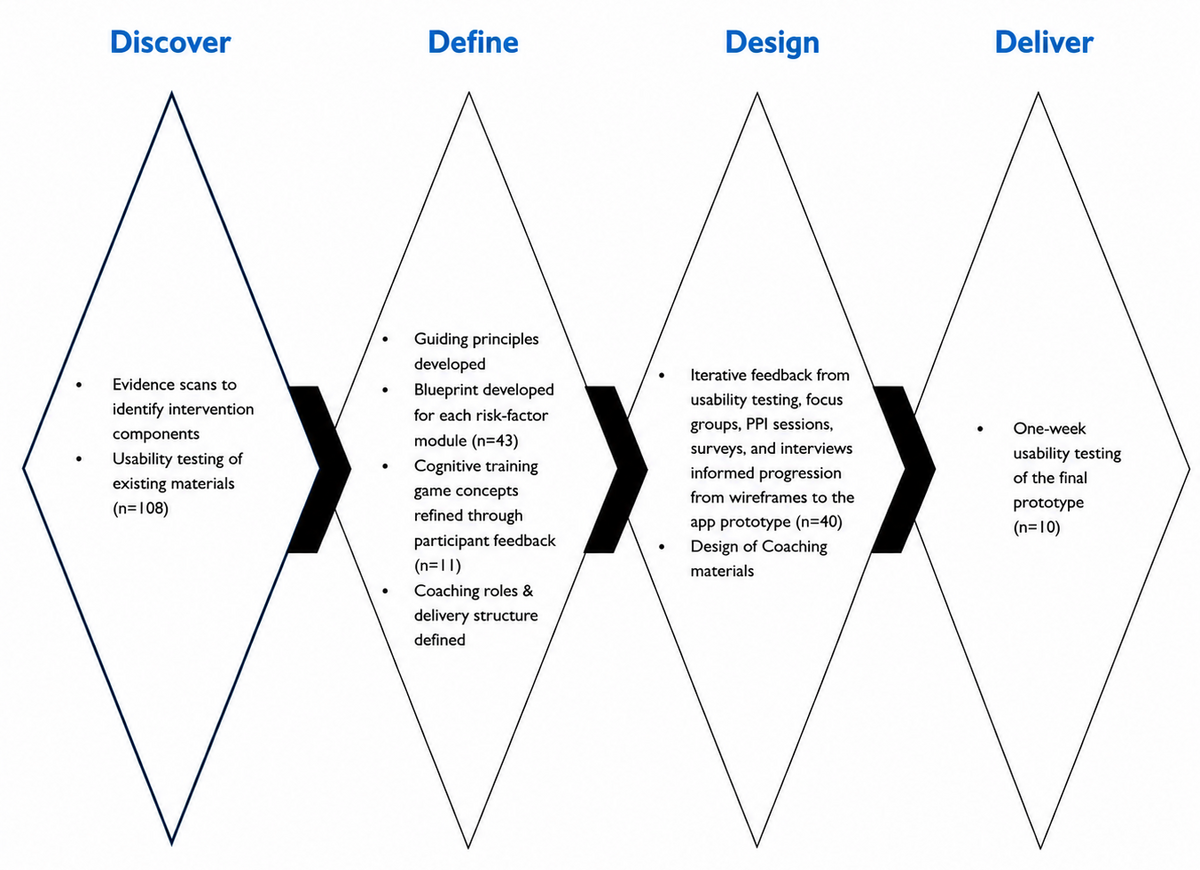
Study flow diagram: the co-design process. PPI: patient and public involvement and engagement.

We drew on established methodological frameworks to structure the development. The Medical Research Council framework for developing complex interventions guided the overall approach [40]. In line with this framework, we developed a logic model at the outset to specify ENHANCE’s core components, proposed mechanisms of action, and expected outcomes (Multimedia Appendix 1). We used the Double Diamond model to structure development activities across the Discovery, Define, Design, and Delivery phases, supporting iterative exploration and refinement of the intervention [41] (Figure 1).

As shown in Figure 1, during the discovery phase, we identified candidate components for each risk-factor module through an evidence scan and conducted usability testing of existing apps and materials to explore how older adults engage with them, informing ENHANCE’s design decisions. The Define phase translated these insights into guiding principles, informed by the person-based approach, to steer key design decisions [37]. This phase also produced detailed blueprints for risk-factor modules, cognitive training games, and coaching structure. During the Design phase, wireframes and prototypes were iteratively refined through usability testing until a stable, high-fidelity version was achieved, and a coaching manual was also designed. The Delivery phase involved a 1-week user-testing period to evaluate the feasibility and usability of the intervention.

The GUIDED (Guidance for Reporting Intervention Development) framework checklist for reporting the development process is provided in Multimedia Appendix 2 [42].

### Stakeholders and Recruitment

Coproduction involved an interdisciplinary research team (clinical academics, risk-factor experts, and app developers), research participants, patient and public involvement and engagement (PPI) contributors, and human–computer interaction (HCI) experts. Research participants and PPI provided lived experience and end-user feedback, while the research team and HCI experts contributed technical, clinical, and design expertise.

Research participants were community-dwelling UK adults aged 50 years or older with no severe impairment affecting basic tablet use. We used purposive and snowball sampling to include underserved communities. The research team identified recruitment venues in London and Staffordshire through food banks, religious centers, community centers serving underserved communities, and neighborhood shopping malls in relatively deprived areas, using the Index of Multiple Deprivation (IMD) [43]. Researchers visited sites, distributed leaflets, and screened interested individuals for eligibility. Additional participants were recruited through existing participants and community center staff.

PPI were adults aged 50 years or older, recruited via researcher networks, participants’ referrals, or prior involvement in dementia prevention research.

HCI experts in user experience and interface design were recruited through the research team’s own professional networks.

### Sample Size

As coproduction primarily used usability testing, sample sizes followed established principles whereby 5-10 users identified approximately 85%-95% of usability problems [44,45]. Both participants and PPI were included in the calculation, as both contributed end-user perspectives as intended users of the app.

For the Discovery phase, 5 participants were required per risk factor area (5 × 9 module materials = 45). For cognitive training games, a larger sample of 50 was predetermined, anticipating 5-10 distinct game types each requiring separate evaluation (5 × 10 games = 50). This gave a total of 95 participants.

For the Define and Design phases, up to 3 iterative cycles were planned per phase, with a minimum of 10 participants per cycle (30 per phase).

For the Delivery phase, 10 participants were predetermined for a single 1-week usability testing iteration, identifying approximately 95% of major usability issues [44].

In the following, we report the coproduction methods according to the Discovery, Define, Design, and Delivery phases of the Double Diamond model.

### Discovery Phase

#### Overview

This phase aims to identify intervention components relevant to each risk factor (through evidence scans) and to understand users’ needs, preferences, and barriers to using existing digital interventions (through usability testing of existing apps and materials).

#### Evidence Scans

##### Overview

We conducted a rapid, pragmatic evidence scan of existing systematic reviews to identify intervention components relevant to each dementia risk factor and inform the design of the ENHANCE risk-factor modules.

##### Paper Search and Inclusion Criteria

We searched MEDLINE (via PubMed), the Cochrane Database of Systematic Reviews, and PsycInfo for systematic reviews and meta-analyses relevant to each dementia risk factor. Searches combined Medical Subject Headings (MeSH) terms and free-text keywords relating to (1) the risk factor, (2) intervention type, and (3) older adults, restricted to English-language systematic reviews or meta-analyses of randomized controlled trials (RCTs). An example search structure for hypertension is provided in Multimedia Appendix 3.

Included papers were peer-reviewed systematic reviews and meta-analyses of RCTs addressing the risk factors, with outcomes related to feasibility, acceptability, or effectiveness in participants aged 60 years or older.

##### Screening and Extraction

A total of 2 researchers (A Spillane and KF) screened titles and abstracts, retrieved full texts, and extracted summary-level conclusions on feasibility, acceptability, and effectiveness.

##### Synthesis and Selection of Component

We synthesized findings per risk factor, then mapped them to their reported outcomes. We reviewed and discussed this overview and shortlisted intervention components for each ENHANCE module based on:

- Evidence of feasibility, acceptability, and effectiveness in older adults, prioritizing interventions with higher completion rates, positive user feedback, and behavioral changes improvements within approximately 6 weeks.
- Compatibility with coach-supported digital app delivery, favoring interventions delivered through app-based content supported by brief, remote coaching.
- Scalability of the interventions, prioritizing automated or self-guided components over sustained professional input
- Suitability for underserved users, favoring interventions with less literacy and technical demands.

Following team discussion, a primary intervention strategy was selected for each risk-factor module, forming the evidence base for each module’s content and structure.

#### Usability Testing of Existing Apps and Videos

##### Overview

Usability testing in this phase explored what works when older adults engage with existing apps and digital materials for dementia risk factors, to guide the development of ENHANCE.

##### App Selection

We began by selecting apps that demonstrate a balance of user engagement and scientific evidence, applying a novel 2D framework (Multimedia Appendix 4) to evaluate and rank apps simultaneously across both dimensions. This approach was motivated by evidence that engagement and scientific rigor are often dissociated in mobile health: popular apps frequently lack clinical validation, while evidence-based apps often show poor real-world engagement [46,47]. Selecting apps strong on both dimensions maximizes the possibility of developing an effective app that participants use.

Multimedia Appendix 4 details the identification and selection procedures for all apps. Briefly, we searched app stores and literature, screened against eligibility criteria, extracted app characteristics, rated and ranked apps on user engagement and scientific evidence, and finalized selection using this data through team discussion.

The following apps were selected for testing:

- Cognitive training games: Lumosity (Lumos Labs), Neuronation (Synaptikon GmbH), Cognifit (Cognifit Inc), and Brain HQ (Posit Science)
- Lifestyle apps: Drink Free Days (National Health Service [NHS]), Smoke Free (23 Ltd), Mood Mission (MoodMission Pty Ltd), and Active 10 (NHS)

##### Data Collection

A total of 4 team members (CTKY, JJ, KF, and A Spillane) conducted 45-minute think-aloud usability sessions with research participants using the selected apps (see Stakeholders and Recruitment section for recruitment details), following a predesigned guideline (Multimedia Appendix 5). Participants completed a brief questionnaire capturing brief demographics and then navigated the app on a tablet while verbalizing their thoughts. Researchers recorded observational notes on navigation paths, difficulties, and pauses, and invited participants to share any general impressions or additional comments at the end.

The same group of team members conducted separate 45-minute semistructured individual interviews with a different participant group to gather feedback on videos and educational materials covering dementia risk factors. Videos included 8 diabetes and hypertension videos and 6 NHS exercise or healthy eating videos. Educational materials included exercise infographics, healthy eating guidelines, and healthy recipes from the NHS and national charities. Participants were asked what they liked, disliked, and how materials could be improved. We audio-recorded all sessions.

##### Data Analysis

Transcripts were analyzed using a reflexive thematic analysis approach [48,49] by the same group of researchers using NVivo (Lumivero). Each researcher independently coded the transcripts; codes were discussed and refined across regular team meetings and with supervisors (SGC and GL) until final themes were agreed upon. Analysis focused on identifying participants’ preferences, barriers, and usability issues, and the support needed. Findings were summarized by risk factor and informed the content and design of the risk-factor modules and cognitive training games.

### Define Phase

The Define phase translated discovery findings into concrete design decisions for ENHANCE.

#### Defining the Guiding Principles

Following the person-based approach, we translated discovery findings into a set of guiding principles to keep the intervention focused, coherent, and user-centered [37,50]. Principles were drafted by integrating the literature, behavior change theory, discovery findings, and the team’s clinical experience, then iteratively refined with coinvestigators and PPI through monthly meetings. They served as decision rules guiding the overall structure and content of the risk-factor modules, coach–participant interactions, and cognitive training games.

#### Defining the Unified Visual Theme and Reward Framework

The research team worked with PPI and participants to define the app’s visual theme and reward structure.

##### Data Collection: Group Interviews

Three 1-hour semistructured facilitated group interview sessions were conducted—1 online session with PPI and 2 in-person sessions with participants—facilitated by trained team members (JJ, KF, CTKY, and A Spillane). Participants viewed options for the app’s visual theme and reward structure and provided feedback. Sessions were audio-recorded.

##### Data Analysis

Given the rapid iterative nature of the coproduction process, feedback was reviewed and synthesized into key points by session facilitators, discussed with the wider team, and shared with the app development company to agree on the final design collaboratively. This approach was applied consistently across all subsequent Define and Design phase activities.

#### Defining Risk-Factor Module Structures and Coaching Roles

This phase aimed to define the structure and content of individual risk-factor modules and associated coaching roles. Module development was informed by the evidence scan, guiding principles, BCT taxonomy [51,52], and relevant clinical guidelines.

The research team, in consultation with domain experts, developed preliminary module blueprints as visual flowcharts outlining the proposed sequencing of activities across 6 weeks, including in-app components and coaching interactions. Blueprints were iteratively refined through expert discussions and a 1-hour PPI group session until consensus. Building on these blueprints, the team developed detailed module specifications outlining weekly in-app features (eg, check-in questions, response logic, tailored messages, and video content) and coaching roles. Specifications were refined with PPI feedback before being translated into wireframes in the Design Phase.

#### Defining Ideas and Concepts for Cognitive Training Games

##### Co-Design Workshop

We conducted a half-day in-person co-design workshop with the app company’s game designer and the core research team, including 3 experienced clinical academics with expertise in dementia prevention and cognitive training (GL, SGC, and HKP) and 4 researchers who had led prior game usability testing (CTKY, KF, A Spillane, and JJ).

Drawing on the guiding principles and the discovery usability findings, the workshop comprised structured brainstorming, collaborative sketching, and group discussion. Each game was mapped to targeted cognitive domains, producing an agreed list of game types mapped to cognitive domains.

##### Data Collection: User Feedback on Game Concepts

Game types were then presented to PPI (a 1-hour online facilitated interview group session, with videos of similar games) and research participants (45-minute in-person individual interview session, with similar games to play) separately, gathering feedback on appeal, clarity, difficulty, and accessibility. All sessions were audio-recorded.

##### Data Analysis and Game Shortlisting

Following each session, CTKY synthesized the feedback and compiled a structured summary capturing key preferences, concerns, and suggestions. Games were shortlisted based on 4 criteria: breadth of cognitive domain coverage, alignment with the predefined guiding principles, engagement and accessibility for the target population, and development feasibility. The app development company then proceeds with the development of these shortlisted games.

### Design Phase

#### Overview

The design phase translated the Define phase’s outputs into an iteratively refined, high-fidelity prototype of the intervention prototype—including the digital app and associated coaching materials—ready for feasibility testing.

#### Design of the Digital App

##### App Design and Wireframing

###### Overview

We conducted 2 sequential activities—focus groups on initial wireframes and iterative think-aloud usability testing on the resulting prototype—to refine the app design and architecture.

###### Data Collection

A total of 2 focus group sessions—one online with PPI and one in-person with research participants (60-90 minutes each)—gathered feedback on initial wireframes covering the app’s overall concept, core functions, and visual design. Subsequently, iterative one-to-one think-aloud usability testing sessions (approximately 45 minutes each) were conducted with a separate group of research participants using the initial clickable prototype, following the same approach as described in the Discovery phase. All sessions were audio-recorded.

###### Data Analysis

The focus group feedback was synthesized after each round, discussed with the app development company, and feasible refinements were incorporated into subsequent iterations until an acceptable prototype was achieved.

##### Video Content Development and User Feedback

###### Overview

In parallel with app development, we sourced suitable videos for each risk factor module from trusted sources (eg, the NHS and registered charities). These videos covered foundational knowledge about each risk factor alongside practical tips for improvement (eg, dietary guidance). Where suitable materials were unavailable, we developed original videos. All videos used simple English narration, subtitles, and clear visuals to ensure accessibility for underserved populations.

###### Data Collection

PPI completed a brief online survey on the videos the team had developed, each commenting on a subset of 43 draft videos across modules to review online. The survey comprised Likert-scale items (1-5) assessing perceived quality, ease of understanding, interest, and length, alongside 3 open-ended questions on what they liked, disliked, and how the videos could be improved.

###### Analysis

Quantitative responses were summarized by calculating mean scores across each module for each aspect. CTKY reviewed the open-ended responses and summarized refinement priorities, discussed them with the wider research team, and the results were then used to guide video revisions.

##### Technical and Expert Evaluation

###### Internal Team Testing

Team members (CL, CTKY, VM, RB, HK, JJ, and LBR) tested each risk-factor module to identify usability issues and technical bugs. All issues were logged in a shared tracker and categorized by severity (ie, high-, medium-, and low-priority). Testing cycles were repeated until high- and medium-priority issues were resolved.

###### Heuristics Evaluation With HCI Experts

A researcher (CTKY) also conducted a 1.5-hour in-person heuristic evaluation session with HCI experts using the high-fidelity game prototype. HCI experts first spent approximately 30 minutes freely exploring all 7 cognitive training games to familiarize themselves with the gameplay and features. They then independently completed a predesigned evaluation form (Multimedia Appendix 6) based on Nielsen’s 10 usability principles [53,54] for each game, documenting usability issues and recommendations against each heuristic. A group discussion followed to clarify findings.

CTKY synthesized the HCI experts’ group and individual feedback, then discussed with the wider team, and shared with the app development company for revisions.

#### Design of the Coaching Component

We developed the coaching structure, coaching manual, and coach training manual through an iterative process of research team meetings. Development was led by 4 junior researchers (CL, VM, KF, and A Spillane) with nonspecialist health care backgrounds similar to those of the intended coaches. They worked alongside senior team members with expertise in behavior change, coaching, and intervention development (JJ, SGC, GL, ES, and PR), who provided oversight and input throughout the process.

#### Intervention-Level BCT Alignment

Following the development of both digital and coaching components, we conducted a final intervention-level check to ensure fidelity to the underlying behavior change framework. Team members cross-checked all intervention features and content (app-based features and coaching components) against the BCT taxonomy. We developed a mapping table linking each intervention component (risk-factor modules, games, app-level features, and coaching) to specific BCTs; this mapping is reported in the Results section.

### Deliver Phase

The Delivery phase aimed to finalize and refine the intervention through real-world use before feasibility testing.

#### Extended 1-Week Usability Testing

##### Overview

A total of 10 research participants aged 60-80 years with at least 1 dementia risk factor were recruited from the existing recruitment pool. Participants used the app at home for over 1 week, supported by a 45-minute coaching onboarding session, 1 brief follow-up (10-minute video or telephone call), and an in-app messaging platform with their coach. Full methods are reported elsewhere [55].

##### Data Collection

Data were collected from 1-hour poststudy interviews exploring experiences of app use and coaching experiences, backend analytics (frequency and duration of app use), in-app coach–participant messages, and an 8-item satisfaction survey assessing usability, content relevance, likelihood of behavior change, and coaching satisfaction using a 5-point Likert scale [55].

##### Data Analysis

Survey and backend analytics data were summarized descriptively. Interview recordings were transcribed verbatim and analyzed using reflexive thematic analysis [48,49] by the lead researcher (CTKY), with themes refined through discussions with supervisors (SGC, GL, AS, and HKP). In-app messages were coded and used to supplement interview findings. Findings were triangulated across data sources to inform refinement priorities, discussed with the app company, and used to guide final revisions.

#### Reviewing the Exit Criteria

At the conclusion of testing, the team reviewed ENHANCE against 5 exit criteria (Multimedia Appendix 7): technical quality, usability, behavioral fidelity, functional stability, and safety and acceptability. Once all criteria were met, the intervention was deemed ready for the feasibility trial.

## Results

### Participants

Participants were recruited across 11 venues in London and Staffordshire, including food banks, community centers (serving individuals experiencing poverty, specific religious groups, and ethnic minority communities), and a shopping center. PPIs were recruited through researcher networks, many of whom had previously taken part in our other aging research projects, while HCI experts were recruited through our professional networks. In total, 199 unique individuals contributed to coproduction activities: 162 research participants, 33 PPIs, and 4 HCI experts. Participation spanned across 4 phases—Discovery (n=108), Define (n=54), Design (n=40), and Deliver (n=10) (Figure 1)—with some individuals contributing to more than 1 phase.

Participants had a mean age of 65 (SD 9, range 50-87) years, and 65% (106/162) were female; detailed demographic characteristics are presented in Table 1. The largest ethnic group was those who identified as Asian (78/162, 48%), followed by White (47/162, 29%), and Black (27/162, 17%). The mean IMD was 3 (SD 2, range 1-9). Most participants lived in socioeconomically deprived areas, with 79% (110/139) in the bottom 40% of IMD areas. Almost half (68/150, 45%) were retired, and over half (66/131, 50%) had not attained qualifications beyond General Certificate of Secondary Education (an exam taken at the minimum school-leaving age in the United Kingdom). English was the first language for around half (71/154, 46%), and only 44% (68/155) of participants stated that they felt comfortable or very comfortable using technology.

Although demographic data were not collected for the PPI or HCI experts, PPIs were generally female, aged older than 50 years, with diverse ethnic and educational backgrounds. HCI experts were primarily PhD students and postdoctoral researchers in their late 20s to mid-30s, specializing in HCI and app development.

**Table 1:** Research participants’ demographics for the development process (n=162). Missing data were observed for some variables: age (5/162, 3%), IMD (24/162, 15%), occupation (12/162, 7%), highest qualification (31/162, 19%), English as first language (8/162, 5%), and technology comfort level (7/162, 4%). Percentages may not sum to 100% due to rounding.

Variable		Value
Age (years), mean (SD); range		65 (9); 50-87
IMD^a^ decile^b^, mean (SD); range		3 (2); 1-9
**Sex, n (%)**		
	Male	56 (35)
	Female	106 (65)
**Ethnicity, n (%)**		
	White	47 (29)
	Asian	78 (48)
	Black	27 (17)
	Mixed	5 (3)
	Any other ethnicity that has not been specified above	5 (3)
**IMD group (based on UK deprivation percentiles)^b^, n (%)**		
	More deprived areas (bottom 40%; IMD 1-4)	110 (79)
	Less deprived areas (Top 60%; IMD 5-10)	29 (21)
**Occupation, n (%)**		
	Managerial or professional	15 (10)
	Never worked or long-term unemployed	9 (6)
	Routine and manual	6 (4)
	Sick or disabled and unable to work	16 (11)
	Retired	68 (45)
	Others	14 (9)
	Full-time home carer	22 (15)
**Highest level of qualification, n (%)**		
	No education at all or entry-level	28 (21)
	GCSE^c^, BTEC^d^, or O level^e^	38 (29)
	A level	28 (21)
	Diploma of higher education	9 (7)
	Bachelor’s degree or above	28 (21)
**Is English your first language? n (%)**		
	No	83 (54)
	Yes	71 (46)
**How comfortable are you using technology? n (%)**		
	Very uncomfortable	37 (24)
	Somewhat uncomfortable	19 (12)
	Neutral	31 (20)
	Somewhat comfortable	50 (32)
	Very comfortable	18 (12)

^a^IMD: Index of Multiple Deprivation.

^b^The decile scale ranges from 1 to 10, where a decile of 1 indicates that the postcode falls within the most deprived 10% of the deprivation index, while a decile of 10 signifies the least deprived 10%.

^c^GSCE: General Certificate of Secondary Education.

^d^BTEC: Business and Technology Education Council.

^e^O level: General Certificate of Education Ordinary Level.

### Discovery Phase: Key Outputs Informing Intervention Design

#### Intervention Strategies Identified From Evidence Scans

Across the evidence scans, we identified a primary intervention strategy for each risk-factor module. An illustrative example of how the evidence scan informed the selection for the hypertension module is provided in Multimedia Appendix 8. Further, Multimedia Appendix 9 presents the intervention strategy selected for each module and the rationale.

#### Insights From Usability Testing of Existing Digital Materials

##### Selecting and Testing Digital Materials and Videos

A total of 108 participants took part in usability testing of existing cognitive training and lifestyle apps, and reviewed videos and materials across targeted dementia risk factors (Multimedia Appendix 10). As no suitable materials were identified for hearing loss or social isolation, content for these modules was developed with domain experts and refined through user feedback.

##### Feedback

Participants found dense text, complex layouts, technical language, and competing visual elements difficult to engage with—particularly those with lower literacy or limited digital experience. Simple interfaces, pictures over texts, and clear navigation were seen as more accessible and motivating. For cognitive training games, participants favored classic, familiar formats that were mentally stimulating yet achievable, with adaptive difficulty and clear instructions and in-game feedback.

Regarding the gamification features of the app, participants valued personal progress indicators and virtual rewards but disliked competitive scoring. Educational videos were preferred when combining practical guidance with relatable personal stories over expert-led or jargon-heavy content.

Personalization was important, with preferences for adjustable font size, contrast, narration speed, and content format. Many participants needed one-to-one support for basic tablet interactions, highlighting the need for structured onboarding and ongoing human assistance.

### Define Phase: Translating Insights Into Intervention Architecture

#### Guiding Principles Informing Intervention Design

The Define phase translated findings from the Discovery phase into a set of guiding principles that shaped all subsequent design decisions. Table 2 summarizes the guiding principles, their sources, design objectives, and resulting intervention features.

**Table 2:** Guiding principles for ENHANCE (Tailored Intervention for Brain Health and Cognitive Enrichment for Cognitive Health) intervention development.

Sources and key findings	Design objective	Intervention features
From the literature, behavior change theory, and usability testing, BCTs^a^ have been shown to improve behavior change outcomes	Integrate evidence-based BCTs in the app and coaching	Goal setting and action planning: joint goal setting with the coach SMART^b^ goals are achievable within 6 weeks Focus on 1 risk factor at a time Self-monitoring and feedback: activity logging with feedback from app and coach Social support: regular coach contact (meetings and messaging) and Family and friend involvement encouraged Knowledge and skills: clear and practical information Step-by-step brief rehearsing (eg, blood pressure monitoring and exercise demonstrations)
From literature, theory, usability testing, and PPI^c^: positive framing sustains behavior change	Use strengths-based and positive reinforcement	Encouragement and rewards Strength-based language Emphasis on short-term gains and long-term benefits Relatable success stories
From literature, PPI, and usability testing: overburden—whether from multiple simultaneous behavior changes or complex app design—undermines engagement and sustained behavior change	Minimize cognitive and digital burden	Single active risk-factor module at a time Sequential progression guided by participant priorities and the coach Module completion before starting next Simple language Clear and simple in-game instructions and feedback Icons and videos prioritized over text A ≤12-year reading level Clear navigation Coach support for basic app use Jargon and text-heavy information avoided
From usability testing: gamification features—including progress tracking, personal rewards, and familiar or classic games with adaptive difficulty—enhanced engagement; competitive scoring relative to others was not preferred	Use of gamification features and familiar or classic games	Unified visual theme Positive reinforcement (messages, stars, and virtual meadow) Classic cognitive games with adaptive difficulty, personal progress rewards only; no competitive leaderboards
From PPI discussions: early, visible improvements are motivating and self-reinforcing	Design for rapid and observable progress	Components with effects within 6 weeks Trackable metrics (eg, blood pressure and steps) Early positive feedback
From PPI discussions: keeping the intervention “in-house” reduces participant and coach burden	Minimize external navigation	Most activities are delivered within the app Clear signposting to essential in-person care (eg, general practitioner and smoking cessation)
From literature, PPI, and usability testing: participants valued personalization, choice, and flexibility, and had heterogeneous physical abilities, needs, and preferences	Promote autonomy and tailor to diverse capabilities	Participant choice of risk factor module order and goals Multiple activity options and difficulty and mobility levels per module (eg, exercise videos) Customizable app settings (eg, sound and volume) One-to-one coaching to address individual needs

^a^BCT: behavior change technique.

^b^SMART: Specific, Measurable, Achievable, Relevant, and Time-Bound.

^c^PPI: patient and public involvement and engagement.

#### Visual Theme and Reward Structure

Participants favored nature-based themes reflecting growth and progress, informing 2 candidate concepts: a Meadow theme (growing flowers) and a Bird theme (raising a bird through activities) (Multimedia Appendix 11). Following review with PPI and research participants, the Meadow theme was selected. Users earn virtual seeds for completing activities, growing their own meadow, and linking task completion to visible progress toward brain health goals.

#### Risk Factor Module Development

The Define phase produced detailed blueprints for each risk-factor module, clarifying coaching roles and content across a 6-week structure with weekly content release. All modules followed three stages: (1) goal setting (week 1): participants set personalized goals with their coach, supported by in-app check-in questions to capture their typical habits and starting point; (2) Self-monitoring (weeks 2-6): participants engaged in app-based tracking and practical video content, with real-time app feedback and tailored coach guidance; and (3) review and feedback (weeks 2-6): coaches reviewed app-generated summaries, explored barriers, and supported problem-solving with participants.

Blueprints specified activity sequencing, interface design, check-in question wording, and the logic underpinning automated app responses. Multimedia Appendix 12 presents the hypertension module as an illustrative example.

#### Cognitive Training Games: Selection of Game Concepts

Participant and PPI feedback informed the shortlisting of games. Of the 9 candidate games, 2 were excluded (Multimedia Appendix 13). The 7 selected games featured intuitive, easy-to-learn gameplay, positive user reception, variation in difficulty, and coverage across a diverse range of cognitive domains (visuospatial working memory, inhibitory control, numerical reasoning, and language). Excluded games were unsuitable due to high literacy demands and a test-like format (Story Recall), or excessive complexity and fine motor demands that limited accessibility (Train of Thought). Where needed, selected games were refined based on feedback—for example, as some participants associated Caterpillar Chase with schoolwork, its framing and language were refined to present it as a fun, story-based challenge, improving its acceptability. Full details and rationale are provided in Multimedia Appendix 13.

#### Coaching Roles and Delivery Structure Development

Another output of the Define Phase was the specification of a structured coaching model designed to complement app-based delivery. Coaches were defined as nonspecialist staff with backgrounds in health care or related fields (eg, health workers or psychology graduates). Their role was to provide personalized behavioral support alongside app use, with a focus on goal setting, problem-solving, and sustained engagement rather than specialist care.

The model was grounded in the “Goal, Reality, Options, and Will” coaching model [56] and the BCT taxonomy [38,51], informing coaching functions, including “Specific, Measurable, Achievable, Relevant, and Time-Bound” goal setting [57], action planning, feedback tailored to participant-reported data and app-generated summaries, barriers identification, and agreeing on actionable next steps.

Coaching comprised an initial onboarding session, followed by fortnightly remote sessions alongside ongoing app use, with in-app messaging available between sessions.

### Design Phase: Development of a High-Fidelity Prototype

#### Overview

The Design Phase produced an iteratively refined, high-fidelity prototype of the ENHANCE app and coaching materials. Key usability findings and design modifications are summarized below.

#### Overall App Structure and Visual Design

Early wireframes feedback supported the meadow-based visual theme, but noted it felt too distant, visually cluttered, and washed out (Multimedia Appendix 11). The design was subsequently refined by zooming in on the meadow view, reducing visual clutter, and brightening the color palette. Participants also responded positively to weekly check-in questions, educational videos, in-app coach messaging, and gamification features such as earning points and virtual rewards, which were used and integrated into the final app structure.

#### Cognitive Training Games: Usability Findings and Refinements

Participants found the 7 shortlisted games engaging and easy to learn, but identified several usability issues. Participants reported that in-game instructions and feedback were insufficient in some games. For example, unclear instructions in the word-based game led some participants to overlook the “tip” function, while limited auditory and visual feedback in the Caterpillar Race game left participants uncertain about response accuracy. In response, clearer instructional cues were added (eg, text highlighting key functions), and feedback mechanisms were strengthened using more salient auditory and visual signals to indicate correct and incorrect responses. Participants also expressed a desire to better understand the relevance of each game to brain health. To address this, brief, lay-language explanations describing the cognitive or brain health benefits of each game were added to introductory screens.

Game-specific refinements were also implemented. For example, the initial difficulty level of Worm Hunter was reduced following reports that entry-level tasks were overly challenging. In Caterpillar Race, usability issues related to the on-screen keypad—such as unfamiliar symbols and uncertainty around response submission—were addressed by replacing ambiguous icons with more intuitive controls, improving visual clarity, and incorporating clearer submission guidance into the onboarding process, where a participant played the game alongside a coach to guide them.

HCI experts’ feedback reinforced these findings and prompted additional refinements, lengthening time limits in more complex games, revealing correct answers to support learning, and adding brief explanations of how each game benefits cognition. These changes were incorporated into subsequent iterations.

#### Risk-Factor Modules: Refinement of Content

Participants responded positively to check-in questions and educational videos in the risk factor modules.

Check-in questions were perceived as easy to answer and supportive of behavior change through follow-up messages. However, some participants reported that language was overly technical and that certain screens were visually complex (eg, uncertainty around blood-pressure logging in the hypertension module). In response, wording was simplified, and on-screen complexity was reduced to improve clarity and usability.

Educational videos received consistently high ratings across modules. Mean ratings (on a 5-point Likert scale) were 4.3 (SD 0.7) for overall quality, 4.3 (SD 0.6) for interest, 4.3 (SD 0.6) for ease of understanding, and 4.2 (SD 0.6) for appropriateness of length. Participants valued videos that combined practical advice with relatable personal stories and clear signposting to NHS services and relevant organizations.

Participants also provided suggestions for content enhancement, including addressing stigma and demonstrating hearing-aid use in the hearing-loss module, and expanding lifestyle guidance related to smoking, salt reduction, and physical activity in the hypertension module. These suggestions informed subsequent video revisions.

Feedback on videos also highlighted accessibility and inclusivity considerations. Participants recommended slower speech, reduced texts, more culturally representative imagery, and greater customization options (eg, toggling subtitles on/off, adjusting volume, and controlling speech speed). In response, visual content was updated to reflect cultural diversity, subtitles were added to all videos, and narration speed was reduced. Multiple language options and further customization features, such as toggling subtitles and adjusting the narration speed of the videos, were not implemented at this stage due to resource constraints; however, simplified language was used throughout to maximize accessibility in the English-language version. These features will be considered for the future.

#### Coaching Materials: Content, Training Package, and Refinements

A further output of the Design Phase was the development of a complete set of coaching materials to support consistent delivery by nonspecialist coaches.

The coaching package included a coaching manual and coach training materials. The coaching manual specified coach roles and responsibilities, core coaching skills, and practical guidance on app set-up and the key topics to be covered during onboarding and follow-up coaching sessions. The coaching training resources covered content aligned with the manuals and incorporated practical demonstrations and role-play exercises to support skill development and confidence in delivery. During the intervention period, coaches also received fortnightly supervision, supplemented by ad hoc consultations for urgent queries.

Following iterative reviews by team members with nonspecialist backgrounds similar to the intended coaches, the coaching materials were refined to improve usability and acceptability. Revisions included simplifying language, condensing content, adding a dedicated frequently asked questions section, incorporating suggested coaching scripts, and providing guidance on responding to participant requests for medical advice.

#### Fidelity to the Behavior Change Framework

A final intervention-level fidelity check confirmed that the ENHANCE intervention incorporated most core BCT categories across both digital and coaching components. The full mapping of intervention features to BCTs is presented in Multimedia Appendix 14.

### Deliver Phase: 1-Week Usability Testing and Final Refinements

#### Overview

The Delivery phase culminated in 1-week usability testing of the near-final, fully functional ENHANCE prototype, which demonstrated high acceptability, usability, and engagement with both app-based and coaching components. A detailed account of the findings from this phase is reported in a companion paper [55]. Briefly, all participants exceeded the minimum expected app activity, reported high satisfaction with both the app and coaching, and indicated they would continue using the app and recommend it to others. Qualitative findings highlighted the cognitive games, personalized coaching, and relatable educational videos as key facilitators of engagement [55].

#### Refinements Informed by 1-Week Testing

##### Overview

Despite overall positive feedback, 1-week testing identified several areas for improvement that informed further refinement of the prototype.

##### Technical Performance

Some participants experienced technical issues during testing, including video streaming delays attributable to large file sizes, occasional failures in coach message delivery, and instances where virtual “seeds” were not awarded upon task completion. These issues were addressed through a combination of software bug fixes, video file size reduction, and upgraded mobile data SIM cards to improve streaming stability. Additionally, the app was configured to automatically predownload and cache video content when a connection was available.

##### Reward and Library Features Clarity

Participants reported not understanding when they received “stars” or “seeds,” and many did not initially notice the library feature for revisiting videos and replaying games. In response, the coaching manual’s frequently asked questions section was updated to clarify reward types, and onboarding procedures were revised to ensure coaches explicitly introduce the library feature. Planned updates include repositioning the library icon for greater visibility and refining the reward system to improve clarity.

##### Customization and Accessibility

Existing customization options, limited to toggling music and sound, were often overlooked due to their placement within the “stars” section. Participants recommended expanding customization to include font size, image size, and color contrast. Planned refinements, therefore, include relocating customization settings to a more prominent location and adding broader accessibility features.

#### Final Prototype

Following refinements, the prototype met all exit criteria (Multimedia Appendix 7): no critical defects, acceptable usability, alignment with the specified BCTs, and stable performance across devices, with no reported safety concerns.

The ENHANCE app was developed on Lenovo Tab M10 and TCL Tab 10 tablets and is compatible with any Android device running an operating system version 10 or above. These devices were selected as an affordable option. The app requires an active internet connection; participants were provided with preconfigured tablets and data SIM cards, so cost did not prevent participation.

The prototype was designed to be UK General Data Protection Regulation (UK GDPR)-compliant, with all participants’ data encrypted and stored on secure cloud servers accessible only to authorized research team members and developers. Participant accounts were secured via 1-time codes sent to registered mobile phones, and coaches accessed the dashboard through 2-factor authentication to protect participant activity data and messages.

Table 3 presents the key features of the final prototype of the ENHANCE app, organized into app-based functions and coach-supported functions. Multimedia Appendix 15 provides screenshots of the app.

**Table 3:** Key features of the ENHANCE (Tailored Intervention for Brain Health and Cognitive Enrichment for Cognitive Health) intervention.

Functions and feature		Goal and function	Description
**App functions**			
	Cognitive training games	Cognitive engagement (RF^a^: low education, but available to all users)	Seven games targeting executive function, attention, memory, language, and numerical reasoning Three new games introduced weekly. Unlocked games remain accessible (up to 10 plays/week) Five rounds per game (approximately 5-10 minutes), with adaptive difficulty
	Check-in questions	Support weekly goal-setting and self-monitoring (RF: all except low education)	1-3 check-ins per week (except hypertension module: 4 readings/day for 4 days)
	Educational videos	Provide practical and educational guidance (RF: all except low education)	Six videos per module (1/week for 6 weeks) Covers condition overviews, personal stories, and practical tips Duration: 2-8 minutes (15-20 minutes for “Getting Active demos”)
	Meadow rewards system	Engagement, gamification, and reinforcement	Home screen navigation Users earn seeds from tasks to plant flowers in their Meadow
	Progress tracker	Visualization of progress	Displays stars earned and flowers planted
	Weekly activity list	Supports habit formation	Shows minimum weekly tasks and completion checkmarks; resets weekly
	Library	Content review	Allows replay of completed games and rewatching of videos that have been unlocked
**Coach-delivered functions**			
	Scheduled coaching sessions	Personalized behavioral support	45-minute in-person onboarding session (to support app learning) plus fortnightly 10-minute phone or video sessions for ongoing support
	Coaching dashboard	Monitoring and tailored support	Coaches access real-time participant activity and progress data
	Messaging Interface	Between-session support	In-app chat allows participants to ask questions and receive coach responses (within 3 working days), enabling ongoing guidance between scheduled sessions
	Optional family involvement (guided by the coach)	Enhance support and motivation	Coaches can help participants involve a family member to reinforce engagement and support behavior change

^a^RF: risk factor.

## Discussion

### Principal Findings

We coproduced ENHANCE, a coach-supported digital intervention targeting modifiable dementia risk factors. This process resulted in an evidence-informed, theory-based, and user-centered intervention designed to reduce dementia risk, with particular attention to accessibility for underserved older adults. By prioritizing accessibility for groups at higher dementia risk—including individuals who are digitally excluded and often underrepresented in prevention research—the intervention was designed to be usable by a broad older adult population. Using a structured, multistage development process, we integrated evidence from the literature, behavioral theories, and coproduction activities with 199 contributors to develop ENHANCE. This approach generated detailed insights into the design and usability needs of underserved groups and informed the development of both a high-fidelity app prototype and a comprehensive coaching package. Together, these components form a complete intervention ready to be tested in forthcoming feasibility and full trials.

### Design Decisions

We developed ENHANCE as an app-based, coach-supported multidomain program targeting 10 dementia risk factors. The intervention includes 7 adaptive cognitive training games accessible to all participants and tailored risk-factor modules. Each module lasts 6 weeks and incorporates educational videos, check-in questions, and “Specific, Measurable, Achievable, Relevant, and Time-Bound” goals to support behavioral change and monitoring. Rewards include visual progress indicators, coach encouragement, and a virtual meadow that participants can plant and grow. Throughout development, scientific evidence, user feedback, and practical considerations informed design decisions.

First, 6-week modules enable early, observable progress and support habit formation, with habit strength typically plateauing within this timeframe [58]. Short-term improvements were designed to enhance motivation and self-efficacy, sustaining behavior change beyond the module period. This was further supported by PPI and participant feedback, emphasizing the value of early visible progress. For risk factors where substantial physiological change within 6 weeks is unlikely (eg, diabetes), modules target achievable self-management behaviors—building skills, establishing routines, and initiating health care contact—placing users on a path toward longer-term change.

Second, we embedded accessibility features throughout ENHANCE, shaped directly by coproduction activities with older adults. This addressed a gap in existing digital interventions where such features are rarely incorporated [27]. We prioritized images over text to reduce reliance on reading ability, avoiding color-dependent game design to accommodate users with color blindness or macular degeneration, subtitled all videos to support hearing impairment, provided adjustable volume, and paired all in-app feedback with both visual and audio cues. Delivering via tablet further supported easier navigation and reduced visual strain.

Cultural accessibility considerations included a nature-based visual theme found to be broadly acceptable across genders and cultures, video content representing diverse ethnicities and religious preferences (eg, food recipes), and exercise options ranging from seated to higher-intensity alternatives. The word bank of a word-based game was curated using culturally universal and commonly understood words. Number-based games were deliberately framed without any reference to “maths,” as feedback from our earlier user testing indicated that such framing was off-putting for some participants.

Digital accessibility considerations included phone number registration in place of email and a simplified login system without repeated password entry. Game design varied in type and difficulty to accommodate diverse educational backgrounds, gaming experience, and physical abilities, with motor demands kept deliberately low. But some accessibility enhancements identified during coproduction—including expanded language options and voice guidance with text-to-speech functionality—could not be realized within the current development scope and remain priorities for future iterations.

Third, we designed each module to address 1 risk factor at a time, supported by early usability testing in which participants preferred focusing on 1 manageable change at a time. This is grounded in the behavioral change model, which posits that tackling multiple changes simultaneously risks exceeding an individual’s capability [38] and the paradox of choice [59], whereby too many options increase cognitive load and decision fatigue. Focusing on a risk factor, therefore, supports clearer goal formation and sustained engagement.

Fourth, ENHANCE was designed to offer tailoring and choice, allowing participants to select which risk factors to address, in what order, and how—for example, choosing specific exercises and frequency. This gave users control and ensured the intervention felt relevant rather than prescriptive, supported by BCT evidence that engagement is greater when individuals choose their own method [38,51]

Fifth, we adopted a front-loaded human coaching model, informed by evidence that digital behavior-change interventions achieve higher engagement when paired with human support [36], and that intensive early contact builds confidence, skills, and familiarity with the intervention, supporting longer-term adoption [60]. The model was also designed to be scalable as coaches were nonspecialists with a variety of health care or related backgrounds, reducing reliance on expensive, highly trained clinicians. The brief fortnightly remote coaching contacts minimized the ongoing coach burden. The in-app messaging made the coach accessible and reduced reliance on scheduled contact. The structured manuals and training materials allow replication and fidelity of delivery. However, whether this coaching model is effective or cost-effective remains to be established through a full RCT, though evidence from similar coaching models in lifestyle interventions suggests potential [61,62].

### Comparing With Existing Dementia Prevention Trials

#### Comparison of Development Processes (Coproduction)

In ENHANCE, end users were partners at every stage of development, contributing to guiding principles, module workflows, games, interface design, and content—a level of ongoing partnership rarely described in the dementia-prevention field.

To our knowledge, only HATICE, PRODEMOS, and Active Brains have published detailed development accounts [50,63,64]. HATICE was developed largely from national cardiovascular guidelines with no reported user involvement [63]. PRODEMOS and Active Brains incorporated iterative input from older adults [50,64], but neither reported key contributor characteristics—such as ethnicity, educational level, socioeconomic position, or digital literacy—leaving it unclear whether underserved groups were meaningfully represented. Without their active involvement, digital interventions risk being less acceptable and less engaging [12].

#### Comparison of Intervention Components

First, ENHANCE was developed specifically with underserved users rarely included in the coproduction of digital dementia-prevention tools. Of 162 participants, the majority lived in highly deprived areas; 71% (115/162) identified as non-White, 50% (66/131) had not been educated beyond the minimum school-leaving age, and only 44% (68/155) felt comfortable using technology. This reflects substantially greater inclusion than existing trials, where ethnic minority participation averages around 25% [10], and samples are typically White, more educated, and more digitally literate [12,65,66].

Second, ENHANCE offers a more intensive coaching structure than comparable interventions. Active Brains limited support to 3 telephone calls and email reminders over 1 year [50], LETHE coaching focused primarily on technical assistance [23], and PRODEMOS and HATICE delivered BCTs largely through online messaging [26,67]. ENHANCE combines face-to-face onboarding, regular remote follow-ups, and in-app messaging using BCTs—supporting digital skill-building, rapport, and real-time troubleshooting, particularly for those from lower socioeconomic backgrounds [36] while retaining the scalability of digital delivery.

Third, ENHANCE targets multiple modifiable dementia risk factors within a single, integrated platform. Programs such as US POINTER and FINGERS commonly use apps for cognitive training while delivering other components through separate formats, whereas a unified platform may offer a more coherent user experience and promote long-term engagement [6,15].

Fourth, ENHANCE features a novel, cohesive gamification approach—a meadow theme symbolizing growth toward better brain health—running consistently throughout the app to reinforce progress and sustain motivation. While PRODEMOS incorporates gamification elements such as points and progress tracking [64,67], it lacks a unified visual or thematic identity, which may limit sustained engagement.

### Usability Findings and Subgroup Consideration

In ENHANCE, we conducted extensive qualitative work with underserved populations to inform the refinement of our app. Our findings broadly align with those reported in the PRODEMOS development work on the digital needs of underserved groups [64]. Our participants highlighted coaching, gamification features, adaptive cognitive training games, and videos as important for initial and sustained engagement and valued positive framing and personalized content. Conversely, complicated navigation, text-heavy interfaces, and overly technical content were perceived as key barriers. This study also adds further insight by suggesting that focusing on 1 risk factor at a time may help prevent user overload and that initially simple, familiar games can support sustained app use. These insights may help guide the design of future digital tools that enhance long-term engagement in underserved groups.

Studies of app-based interventions frequently identified negative attitudes toward technology and privacy concerns as barriers to engagement among older people or underserved populations [36,68,69]. Notably, such themes were absent from our usability testing findings. This may reflect the trust cultivated through in-person contact with a consistent coach and credible research team, or growing societal familiarity with app-based technologies [70], though it is also possible that our think-aloud methodology, which directed participants’ attention toward specific interface interactions, made broader attitudinal concerns less likely to surface.

While underserved groups shared common preferences and barriers, coproduction revealed meaningful differences across subgroups. Ethnic minority participants more frequently requested culturally matched content. For example, broadening dietary recommendations to include vegetarian recipe options. Those from lower socioeconomic backgrounds prioritized affordability, addressed by ensuring all food and exercise content featured budget-friendly and equipment-free options. These findings illustrate a broader principle: designing explicitly for underserved subgroups often improves the intervention for all.

### Lessons Learned From Coproduction

Our coproduction process generated several practical lessons. First, in our coproduction activities, communications between participants and developers were mostly indirect—participant feedback was gathered by researchers and then relayed to the app developer. While this ensured a structured information flow, it could introduce delays and occasional misunderstandings. Direct involvement of users in coproduction workshops would likely strengthen user-centered design by enabling real-time discussion of user needs, technical constraints, and feasible solutions. Second, obtaining meaningful early-stage feedback was challenging when designs were presented abstractly, particularly for less digitally experienced users—a difficulty also noted in PRODEMOS [64]. This improved markedly once clickable prototypes were introduced, enabling richer and more actionable feedback. Third, establishing clear guiding principles at the outset helped anchor the project’s direction, serving as a consistent reference point and reducing design drift. Finally, systematic documentation—including structured records of feedback, design decisions, changes implemented, and changes deferred with reasons—was essential for maintaining clarity and accountability. A shared online platform accessible to all stakeholders, using plain language throughout, supported inclusive decision-making.

### Strengths

The ENHANCE study has several strengths. First, development was theory-driven and evidence-based, with key decisions—including intervention approaches, module cadence, and cognitive games selection—underpinned by robust evidence and user needs. Second, the development was systematic—beginning with a program theory and logic model, followed by evidence scans and usability testing of existing digital materials, with content developed iteratively from resulting design principles. Third, extensive coproduction involved 162 end users from underserved backgrounds, a level of inclusion rarely achieved in digital dementia-prevention research. Fourth, ENHANCE combines scalable app-based delivery with personalized coach support, a model particularly effective for socioeconomically disadvantaged users who may face digital exclusion [36].

### Limitations

This study also has limitations. First, most research participants were recruited in London and Staffordshire, therefore involving few people living in rural areas, limiting the generalizability of our findings. Second, we conducted a rapid, pragmatic evidence scan rather than a full systematic review to identify intervention components for each dementia risk factor module. Therefore, some relevant interventions may have been overlooked.

Third, the app is currently available only on tablets—a deliberate design choice to improve usability, but one that limits accessibility, given that mobile phones are more commonly owned. Although tablets were provided during this study, this may pose challenges for real-world implementation. Future iterations should explore a responsive design approach that adapts across devices, while still maintaining accessibility for older adults.

Fourth, the app is currently available only in English, and during usability testing, participants expressed a desire for versions in other languages (eg, Hindi). While we used simplified language and minimized text to support users with lower literacy, individuals with no English literacy may still face barriers.

Fifth, some design decisions were influenced by practical constraints rather than evidence or user preference alone. Development timelines and technical limitations meant not all coproduction suggestions could be incorporated. For example, a full redesign of the reward system and repositioning of the library icon. These refinements will be prioritized.

Sixth, a formal accessibility audit against a recognized standard, such as Web Content Accessibility Guidelines (WCAG) 2.1 AA [71], was not conducted during development. Instead, we adopted an empirical, user-centered approach, iteratively identifying and resolving usability barriers through repeated testing with users with low digital literacy, guided by HCI expert collaborators—an approach well-suited to surfacing real-world barriers that a standardized checklist might miss. A Google Accessibility Scanner audit confirmed satisfactory touch target sizes, clickable items, contrast ratios, and content labeling, though some interface texts were not fully exposed to screen readers. A formal WCAG 2.1 AA audit will be prioritized in future iterations.

Finally, video streaming delays caused by large file sizes over mobile data connections were identified during testing and addressed iteratively through video compression, SIM card upgrades, and configuring the app to predownload and cache content when connected to the internet. However, mobile data costs and limited offline functionality may present barriers to wider implementation. Future iterations will prioritize minimizing data usage and enabling offline access from the outset.

### Next Steps

Despite the structured development process, uncertainties remain regarding engagement and adherence beyond the 1-week testing period, coach workload in routine practice, and sustainability of behavioral change postintervention. These will be examined in our upcoming feasibility trial, assessing 3-month engagement, usability, and preliminary efficacy, and subsequently in the definitive RCT with embedded process evaluation.

While our coaching model was designed with scalability in mind, human coaching presents challenges for cost-effective deployment at scale. We intentionally limited coach contact time and included only 1 face-to-face meeting, balancing cost-effectiveness with accessibility for users. Cost-effectiveness will be formally examined in the RCT. Given the substantial economic burden of dementia, even a modest effect from a relatively low-cost intervention could prove cost-effective.

In the longer term, AI integration may reduce human burden while maintaining intervention quality. We have already automated lower-intensity tasks—such as reminders, progress summaries, and tailored in-app feedback—while retaining human contact for feedback, motivational support and goal setting. While evidence on AI coaching remains mixed [72,73], incorporating empathy, personalization, and behavior change theory may improve its effectiveness. Future research could explore hybrid human–AI models that progressively reduce human burden without compromising engagement or outcomes [73].

### Conclusion

The ENHANCE intervention was developed using an evidence-informed, theory-driven, and coproduced process to ensure that its design aligned with the needs, preferences, and capabilities of underserved midlife and older adults who are at higher risk of dementia but often underrepresented in prevention research. By involving 162 end users throughout every stage of development, ENHANCE represents, to our knowledge, one of the most comprehensively coproduced digital dementia-prevention interventions to date. The resulting app uses a unified meadow-themed design and incorporates cognitive training games alongside tailored dementia risk factor modules delivered through educational videos and weekly check-in questions. Our usability testing demonstrated high satisfaction, strong engagement, and the feasibility of the intervention. ENHANCE is now ready to progress to a feasibility trial, and if successful, a definitive RCT to assess sustained engagement, implementation feasibility, and its potential to reduce dementia risk at scale.

## Data Availability

The data generated and analyzed during this study are primarily qualitative and include detailed participant accounts produced through co-production activities. Owing to the risk of reidentification, these data cannot be meaningfully anonymized and are therefore not publicly available. Access to selected, redacted extracts may be considered by the corresponding author on reasonable request, subject to ethical approval and data governance constraints.

## Appendix

Multimedia Appendix 1 Initial logic model of ENHANCE.

Multimedia Appendix 2 The Guidance for Reporting Intervention Development (GUIDED) Framework.

Multimedia Appendix 3 Example search strategy for hypertension.

Multimedia Appendix 4 Detailed procedure and results of app selection for usability testing.

Multimedia Appendix 5 Example of think-Aloud Usability Testing Interview Guide for Selected Apps (Lumosity).

Multimedia Appendix 6 Heuristics Evaluation workbook.

Multimedia Appendix 7 Exit criteria for development of app and assessment methods.

Multimedia Appendix 8 Result of evidence scan leading to primary intervention (Hypertension module).

Multimedia Appendix 9 Primary intervention strategy for each risk factor module in ENHANCE.

Multimedia Appendix 10 Participants' demographic characteristics of usability testing of existing digital materials.

Multimedia Appendix 11 Iterative Design of ENHANCE From User Feedback to Final Interface.

Multimedia Appendix 12 Example of the initial hypertension workflow chart.

Multimedia Appendix 13 Co-design of cognitive training games: concepts, user feedback, and selection decisions.

Multimedia Appendix 14 Mapping of behavioural Change techniques to the ENHANCE intervention.

Multimedia Appendix 15 Screenshots of the app.

## Abbreviations

BCT: behavior change technique
ENHANCE: Tailored Intervention for Brain Health and Cognitive Enrichment for Cognitive Health
GUIDED: Guidance for Reporting Intervention Development
HCI: human–computer interaction
IMD: Index of Multiple Deprivation
MeSH: Medical Subject Headings
NHS: National Health Service
NIHR: National Institute for Health and Care Research
PPI: patient and public involvement and engagement
RCT: randomized controlled trials
UK GDPR: UK General Data Protection Regulation
WCAG: Web Content Accessibility Guidelines
