# Supplementary material for: ENHANCE (Tailored Intervention for Brain Health and Cognitive Enrichment for Cognitive Health), a Coach-Supported Digital Intervention for Dementia Prevention in Underserved Older Adults: Co-Design and Usability Study
Source: J Med Internet Res. 2026 Jul 29;28:e91999. doi: 10.2196/91999 (PMC13469833; doi:10.2196/91999)
Supplement: Supplementary file 1 [file jmir_v28i1e91999_app1.docx]

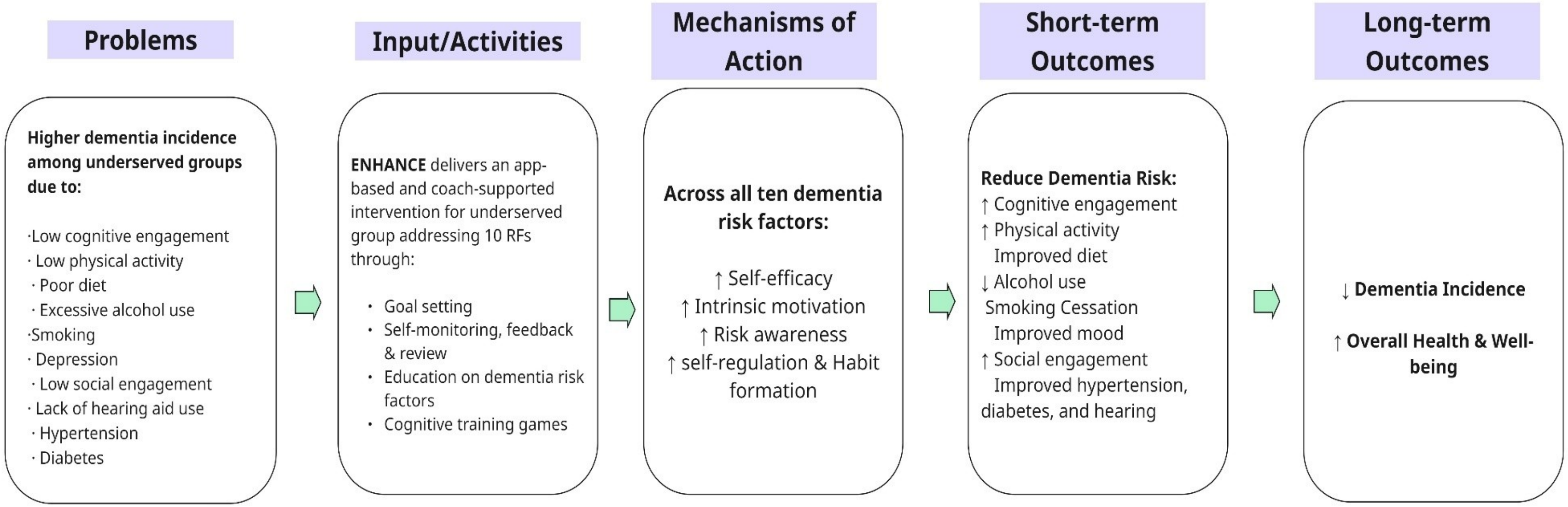
Problems
Input/Activities
Mechanisms of Action
Short-term Outcomes
Long-term Outcomes
Higher dementia incidence among underserved groups due to:
·Low cognitive engagement
· Low physical activity
· Poor diet
· Excessive alcohol use
·Smoking
· Depression
· Low social engagement
· Lack of hearing aid use
· Hypertension
· Diabetes
ENHANCE delivers an app-based and coach-supported intervention for underserved group addressing 10 RFs through:
• Goal setting
• Self-monitoring, feedback & review
• Education on dementia risk factors
• Cognitive training games
Across all ten dementia risk factors:
↑ Self-efficacy
↑ Intrinsic motivation
↑ Risk awareness
↑ self-regulation & Habit formation
Reduce Dementia Risk:
↑ Cognitive engagement
↑ Physical activity
Improved diet
↓ Alcohol use
Smoking Cessation
Improved mood
↑ Social engagement
Improved hypertension, diabetes, and hearing
↓ Dementia Incidence
↑ Overall Health & Well-being
