# Supplementary material for: ENHANCE (Tailored Intervention for Brain Health and Cognitive Enrichment for Cognitive Health), a Coach-Supported Digital Intervention for Dementia Prevention in Underserved Older Adults: Co-Design and Usability Study
Source: J Med Internet Res. 2026 Jul 29;28:e91999. doi: 10.2196/91999 (PMC13469833; doi:10.2196/91999)
Supplement: Supplementary file 2 [file jmir_v28i1e91999_app11.docx]

Examples of Iterative Design of ENHANCE: From User Feedback to Final Interface

1. Based on participants' preference for nature-themed visuals, two routes were co-designed for their selection: **the Bird Route and the Meadow Route**

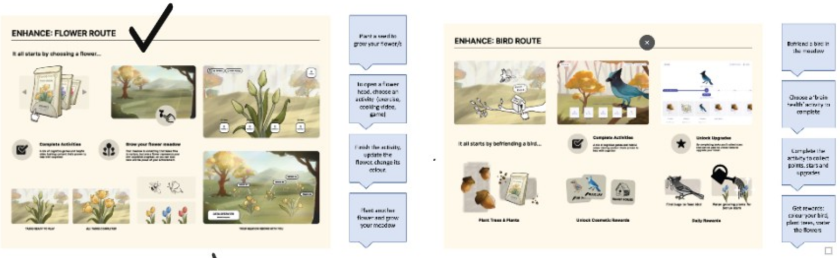

1. The **Flower Meadow Route** was preferred by most participants

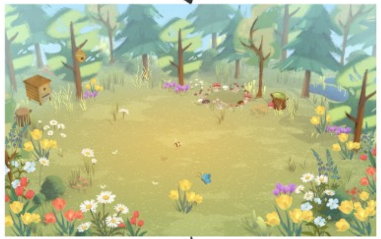

3.Initial design:

- Meadow view too distant
- Crowded screen elements
- Warm, golden tones appearing washed out

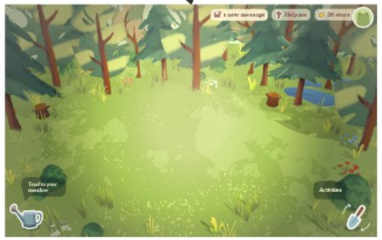

4.Revised design:

- The zoomed-in view makes touch targets larger and easier to tap
- Less visual noise
- Brighter colours are easier to see

1. **Participants' feedback on Initial design (Library) :** Too many items on screen at once

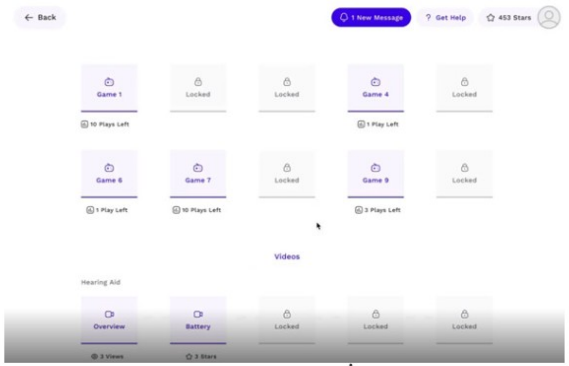

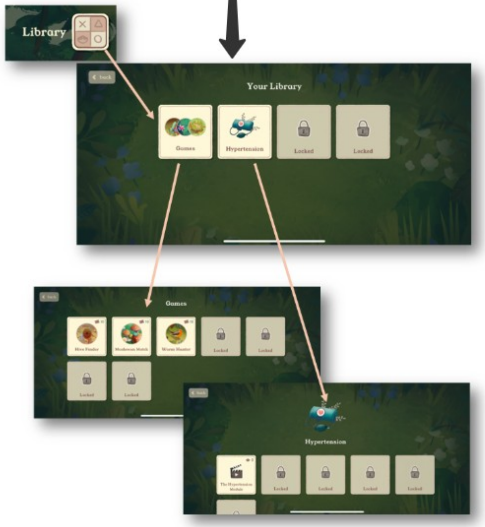

2.Revised Design: Hierarchical category navigation — fewer items per screen.
