# Supplementary material for: ENHANCE (Tailored Intervention for Brain Health and Cognitive Enrichment for Cognitive Health), a Coach-Supported Digital Intervention for Dementia Prevention in Underserved Older Adults: Co-Design and Usability Study
Source: J Med Internet Res. 2026 Jul 29;28:e91999. doi: 10.2196/91999 (PMC13469833; doi:10.2196/91999)
Supplement: Supplementary file 2 [file jmir_v28i1e91999_app10.docx]

## Participants' demographic characteristics of usability testing of existing digital materials

| **Please note that the 108 participants involved in usability testing were a subset of the 162 research participants reported in Table 1 in the main manuscript**  **Participants' demographic characteristics of usability testing of existing digital materials (N=108)** | | | |
| --- | --- | --- | --- |
|  |  | **Mean (SD) (Range)** | |
| **Age** | | 66 (9) (50-87) | |
| **Index of Multiple Deprivation Decile (IMD)*** | | 3 (2) (1-9) | |
|  |  | **N** | **(Valid %)** |
| **Sex** |  |  |  |
|  | Male | 38 | 35 |
|  | Female | 70 | 65 |
|  |  |  |  |
| **Ethnicity** | |  |  |
|  | White | 38 | 35 |
|  | Asian | 45 | 42 |
|  | Black | 20 | 19 |
|  | Mixed | 2 | 2 |
|  | Any other ethnicity which has not been specified above | 3 | 3 |
|  |  |  |  |
| **Occupation** | |  |  |
|  | Managerial/Professional | 13 | 12 |
|  | Never worked/Long term unemployed | 8 | 7 |
|  | Routine & manual | 4 | 4 |
|  | Sick/Disabled & unable to work | 13 | 12 |
|  | Retired | 55 | 51 |
|  | Others | 7 | 7 |
|  | Full time Home Carer | 8 | 7 |
|  |  |  |  |
| **Highest Level of qualification †** | |  |  |
|  | No education at all/entry level | 15 | 16 |
|  | GCSE/BTEC/Olevel | 31 | 32 |
|  | A level | 20 | 21 |
|  | Diploma of higher education | 6 | 6 |
|  | Degree with honours or above | 24 | 25 |
|  |  |  |  |
| **Is English your first language?** | |  |  |
|  | No | 58 | 54 |
|  | Yes | 49 | 46 |
|  |  |  |  |
| **How comfortable are you using technology? †** | | |  |
|  | Very uncomfortable | 24 | 23 |
|  | Somewhat uncomfortable | 14 | 13 |
|  | Neutral | 18 | 17 |
|  | Somewhat comfortable | 33 | 31 |
|  | Very comfortable | 16 | 15 |
| *Participants’ Index of Multiple Deprivation (IMD) was determined using the IMD postcode search tool. The IMD decile is the official measure of relative deprivation in small areas of England, ranging from 1 (most deprived 10%) to 10 (least deprived 10%). | | | |
| † Missing data: One participant was missing data on age and English as a first language (1%). Three participants were missing data on comfort with using technology (3%). Twelve participants were missing data on IMD and highest level of qualification (11%). | | | |
| 108 people participated in 116 usability testing session. The number of tests for each type of material related to dementia risk factors was as follows: cognitive training (4 apps; n = 64), hypertension and diabetes (videos; n = 5), physical inactivity (app, videos, and infographics; n = 8), obesity (recipes; n = 2), depression (app; n = 22), smoking (app; n = 9), and drinking (app; n = 6). | | | |
