# Supplementary material for: ENHANCE (Tailored Intervention for Brain Health and Cognitive Enrichment for Cognitive Health), a Coach-Supported Digital Intervention for Dementia Prevention in Underserved Older Adults: Co-Design and Usability Study
Source: J Med Internet Res. 2026 Jul 29;28:e91999. doi: 10.2196/91999 (PMC13469833; doi:10.2196/91999)
Supplement: Supplementary file 5 [file jmir_v28i1e91999_app13.docx]

| **Game** | **Game concept** | **User feedback** | **Selection decision and rationale** |
| --- | --- | --- | --- |
| **What Word** | Players unscramble letters to form a word, supported by a picture clue. | Positive; familiar, simple, and easy to engage with. | **Selected:** Strong positive reception; intuitive classic format; only game targeting language skills. |
| **Night Sorter (Card Game)** | Players view a sequence of picture cards and identify matches with current cards. | Positive; familiar and easy to learn, though some found it too easy. | **Selected:** Included to provide easier options for users less familiar with gaming; strong positive feedback. |
| **Hive Finder (Card Game)** | All cards remain face-up; players identify and match identical pictures, with increasing difficulty. | Positive; familiar and easy to learn. | **Selected:** Strong positive reception; intuitive format; one of the few games targeting inhibitory control. |
| **Mushroom Match (Card Game)** | Players flip cards to memorise positions and match pairs, with increasing difficulty. | Positive; familiar, fun, and easy to learn. | **Selected:** Targets visual working memory; suitable for lower literacy and limited mobility; classic format. |
| **Stump Shuffle (Card Game)** | Players identify an “odd card,” which is reshuffled, and must locate it again. | Positive; easy to learn and engaging. | **Selected:** Targets visual working memory; minimal motor demands; strong user acceptance. |
| **Worm Hunter** | Players memorise rock positions and draw a safe route for a bird to reach a worm. | Mixed; some enjoyed the challenge, others found it unfamiliar. | **Selected:** Provides greater challenge; only game targeting visuospatial working memory. |
| **Caterpillar Chase** | Players solve numerical problems to stop a caterpillar eating an apple. | Mixed; some found it fun, others felt it resembled schoolwork (feedback based on static designs). | **Selected:** Ensures coverage of numerical reasoning; terminology adjusted to improve acceptability. |
| **Story Recall** | Users read short passages and answer questions immediately or after a delay. | Mixed; content appreciated, but some perceived it as test-like. | **Excluded:** Heavy reliance on language and literacy; test-like format reduced engagement. |
| **Train of Thought** | Players guide multiple trains to stations simultaneously, dividing attention across routes. | Mixed to negative; exciting for some but confusing and motor-demanding for many. | **Excluded:** High complexity and fine motor demands limited accessibility; focus was on cognitive rather than motor skills. |

**Co-design of cognitive training games: concepts, user feedback, and selection decisions**
