# Supplementary material for: ENHANCE (Tailored Intervention for Brain Health and Cognitive Enrichment for Cognitive Health), a Coach-Supported Digital Intervention for Dementia Prevention in Underserved Older Adults: Co-Design and Usability Study
Source: J Med Internet Res. 2026 Jul 29;28:e91999. doi: 10.2196/91999 (PMC13469833; doi:10.2196/91999)
Supplement: Supplementary file 6 [file jmir_v28i1e91999_app14.docx]

## Mapping of behavioural Change techniques to the ENHANCE intervention

|  | | | | | | | | | |
| --- | --- | --- | --- | --- | --- | --- | --- | --- | --- |
| **BCTs** | **Managing high blood pressure** | **Managing high blood sugar** | **Quitting smoking** | **Better hearing** | **Getting active** | **Healthy eating** | **Drinking less** | **Meeting people** | **Better Mood** |
|  | ***(Goal: Learn to manage blood pressure)*** | ***(Goal: Learn to manage blood sugar level)*** | ***(Goal: Quit smoking)*** | ***(Goal: Increase awareness of hearing impairment)*** | ***(Goal: Increase physical activity)*** | ***(Goal: Increase healthier eating habits)*** | ***(Goal: Reduce alcohol Consumption)*** | ***(Goal: Increase meaningful social interactions)*** | ***(Goal: Learn to use behavioural activation for mood management)*** |
| **1. Goals and Planning** | Coach: Agreed on goals (e.g. Replace one salty snack with a fruit) for each session, encourage planning, and review goals | Coach: Agreed on goals (e.g. made an appointment with GP to discuss treatment plan) for each session, encourage planning, and review goals | Coach: Set goals (e.g. stay smoke-free for the first 24 hours) for each session, encourage planning, and review goals  Check-in question, Week 1: User sets a quit date for smoking | Coach: Set goals (e.g. Made an appointment with the audiologist) for each session, encourage planning, and review goals | Coach: Set goals (e.g. go for a walk after dinner three times this week) for each session, encourage planning, and review goals | Coach: Set goals (e.g. cooked healthy meals at least 2 days this week) for each session, encourage planning, and review goals  Check-in: Selects reasons for reducing weight | Coach: Set goals (e.g. at least 2 alcohol free days this week) for each session, encourage planning, and review goals  Check-in: Selects reasons for drinking less | Coach: Set goals (e.g. meet at least 1 person this week) for each session, encourage planning, and review goals | Coach: Set goals (e.g. Do at least 1 activity you enjoy each day) for each session, encourage planning, and review goals |
| **2. Feedback & Monitoring** | Check-in: Log daily BP; the app gives feedback and next-step advice via a traffic light system. Coach: Review participant goals weekly and acknowledge progress | Check-in: Weekly—ask if the user sought GP advice about blood sugar. The app reinforces the behavior with positive feedback if yes, or prompts action if not. Coach: Review participant goals weekly and acknowledge progress | Check-in: Log cigarettes use weekly; the app provides feedback and encourages progress toward quitting. Coach: Review participant goals weekly and acknowledge progress | Check-in: Ask weekly if users have completed key steps for managing hearing loss (GP visit, audiologist, hearing aids). Provide positive feedback if yes.  Coach: Review participant goals weekly and acknowledge progress | Check-in: Ask users weekly to log planned exercise activities (type and date) for the coming week to support monitoring.  Coach: Review participant goals weekly and acknowledge progress | Check-in: Ask users weekly to plan healthy-eating days for the coming week.  Coach: Review participant goals weekly and acknowledge progress | Check-in: Ask users weekly to plan drink-free days (dates) for the coming week.  Coach: Review participant goals weekly and acknowledge progress | Check-in: Ask users weekly to log social interactions from the past week; provide positive feedback when logged.  Coach: Review participant goals weekly and acknowledge progress | Check-in: Ask users to rate their mood after completing activities that improve mood to facilitate monitoring.   Coach: Review participant goals weekly and acknowledge progress |
| **3. Social Support** | Coach support  Family support – coach involves family if the user agrees. | *(Same as the 1st column)* | *(Same as the 1st column)* | *(Same as the 1st column)* | *(Same as the 1st column)* | *(Same as the 1st column)* | *(Same as the 1st column)* | *(Same as the 1st column)* | *(Same as the 1st column)* |
| **4. Shaping Knowledge** | Video: Causes of high BP, BP monitoring, health tips | Video: Causes of high BS, measuring BS, health tips | Video: Smoking triggers and coping strategies | Video: Causes of hearing loss, hearing aid benefits | Video: Exercise demos and health tips | Video: Causes of obesity, healthy eating tips | Video: Alcohol risks and reduction tips | Video: Benfits of socialising and how to connect | Video: Causes of low mood and using BA to improve it |
| **5. Natural consequences** | Video: Covers benefits and positive outcomes of behaviour change. | *(Same as the 1st column)* | *(Same as the 1st column)* | *(Same as the 1st column)* | *(Same as the 1st column)* | *(Same as the 1st column)* | *(Same as the 1st column)* | *(Same as the 1st column)* | *(Same as the 1st column)* |
| **6. Comparison of Behaviours (e.g. Demonstrate the behaviour to users and highlight others' successful performances)** | Video: How to measure BS and a success story on BP control. | Video: Success story on managing BS | Video: Success story on quitting smoking. | Video: Success story on managing hearing loss with a hearing aid. | Video: Guided exercise demonstrations and success story on becoming more active. | Video: Healthy cooking demonstrations and success story on weight loss. | Video: Success story on reducing alcohol consumption. | Video: Success story on improving social connections | Video: Success story on using BA to improve mood. |
| **7. Associations (Providing Rewards, Remove access to rewards)** | Coach: Acknowledge progress when weekly goals are met. Check-in: Provide positive feedback when desired behaviors are reported in-app | *(Same as the 1st column)* | *(Same as the 1st column)* | *(Same as the 1st column)* | *(Same as the 1st column)* | *(Same as the 1st column)* | *(Same as the 1st column)* | *(Same as the 1st column)* | *(Same as the 1st column)* |
| **Repetition & Substitution (reinforcing behaviors or substitute with better habits)** | Check-in: Prompt daily BP logging to build monitoring habits.  Video: Share tips on replacing unhealthy foods with healthier options.  Coach: Practice BP measurement during onboarding; reinforce healthy behaviors with positive encouragement. | Check-in: Provide positive feedback when users report discussing blood sugar with their GP   Video: Share tips on replacing unhealthy foods with healthier options.  Coach: Reinforce healthy behaviors (e.g., eating diabetes-friendly meals) with positive feedback. | Check-in: Provide positive prompts acknowledging smoke-free efforts to encourage repetition.  Video: Share tips on replacing smoking with healthier alternatives.   Coach: Reinforce smoke-free behavior with positive feedback. | Check-in: Acknowledge GP or audiologist visits and hearing aid use with positive prompts to encourage repetition.  Coach: Reinforce hearing aid use and related behaviors with positive feedback. | Check-in: Provide in-app prompts acknowledging users who reported exercising in the past week, encouraging repetition.  Video: Share tips on replacing inactive habits with active ones  Coach: Reinforce exercise and other healthy behaviors with positive feedback. | Check-in: Provide in-app prompts acknowledging users who reported healthy eating in the past week, encouraging repetition.  Video: Share tips on replacing behaviors that lead to unhealthy eating   Coach: Reinforce healthy eating behaviors with positive feedback. | Check-in: Provide in-app prompts acknowledging users for drink-free days, encouraging repetition.  Video: Share tips on replacing alcohol use with alternative activities.  Coach: Reinforce drink-free behaviors with positive feedback. | Check-in: Provide in-app prompts recognizing users for social interactions in the past week, encouraging repetition.  Video: Share tips on replacing loneliness with social activities.  Coach: Reinforce social engagement with positive feedback. | Check-in: Allow users to rate mood after each activity to reinforce the link between activity and improved mood.  Video: Share tips on replacing behaviors that maintain low mood.  Coach: Reinforce engagement in recommended activities with positive feedback. |
| **9. Comparison of outcomes: Information from credible sources, including pros and cons.** | Video: Share health information and tips from credible source.   Coach: Guide users to identify and compare the pros and cons of behavior change | *(Same as the 1st column)* | *(Same as the 1st column)* | *(Same as the 1st column)* | *(Same as the 1st column)* | *(Same as the 1st column)* | *(Same as the 1st column)* | *(Same as the 1st column)* | *(Same as the 1st column)* |
| **10. Rewards & threats (Material or social reward)** | Check-in: Provide in-app prompts acknowledging users for achieving weekly lifestyle goals.  Coach: Acknowledge progress on weekly goals and highlight long-term health benefits | *(Same as the 1st column)* | *(Same as the 1st column)* | *(Same as the 1st column)* | *(Same as the 1st column)* | *(Same as the 1st column)* | *(Same as the 1st column)* | *(Same as the 1st column)* | *(Same as the 1st column)* |
| **11.. Regulations (Help regulate their behaviours & emotions)** | Coach: Discuss with users ways to regulate stress and routine to support consistent BP monitoring and medication use.  Video: Provide information on regulating BP through medication adherence and lifestyle management. | Coach: Support users in regulating diet and stress to maintain stable blood sugar levels.  Video: Provide information on using diabetes medication to help regulate blood sugar. | Coach: Help users regulate cravings and emotions through coping strategies and routines.  Video: Promote nicotine replacement or prescribed medication to support craving regulation. | Coach: Support users in regulating challenges in wearing hearing aids and emotional responses related to hearing loss. | Coach: Encourage users to regulate motivation, and routine to maintain regular exercise. | Coach: Help users regulate eating habits and emotional triggers for unhealthy eating. | Coach: Support users in regulating urges and emotional triggers that lead to drinking. | Coach: Help users regulate social anxiety and build confidence to engage with others. | Coach: Help users identify ways to regulate emotions and stay engaged in activities. |
| **12. Antecedents (Focus on managing conditions before a behaviour occurs by reconstructing the physical and social environment and avoiding exposure to cues)** | Video: Provide tips on adjusting the environment (e.g., reducing salt at home, keeping a BP monitor visible).  Coach: Help users identify and overcome barriers to regular monitoring, healthy eating, and exercise. | Video: Share tips on keeping healthy snacks visible and limiting sugary foods at home.  Coach: Support users in overcoming barriers to maintaining healthy eating routines. | Video: Offer tips on avoiding smoking triggers (e.g., pubs, certain social settings).  Coach: Discuss barriers to staying smoke-free and suggest coping strategies. | Video: Share tips on making environments hearing-friendly   Coach: Support users in overcoming barriers to wearing hearing aids | Video: Show ways to create a movement-friendly environment (e.g., walking rather than taking lifts).  Coach: Help users plan ways to overcome barriers to exercise. | Video: Provide tips on creating a healthy food environment (e.g., avoiding sugary snacks at home).  Coach: Address barriers to healthy meal prep or grocery shopping. | Video: Share tips on avoiding drinking triggers (e.g., pubs, keeping alcohol out of sight).  Coach: Help users manage social or emotional triggers for drinking. | Video: Encourage creating opportunities for connection (e.g., joining local groups).  Coach: Support users in overcoming social anxiety or logistical barriers. | Video: Provide tips on setting up a positive environment (e.g., developing routines).  Coach: Help users identify and plan around barriers to activity engagement. |
| **13. Identity (Create or change a person's identity related to behaviour)** | Coach: Help users identify as individuals who practice desired behaviours, highlight strengths, and encourage them to model these behaviors for others. | *(Same as the 1st column)* | *(Same as the 1st column)* | *(Same as the 1st column)* | *(Same as the 1st column)* | *(Same as the 1st column)* | *(Same as the 1st column)* | *(Same as the 1st column)* | *(Same as the 1st column)* |
| **14. Scheduled consequences (Remove the reward)** | Check-in: No positive prompts if BP not logged; alert users and provide advice if readings are outside the normal range. | Check-in: No positive prompts if the user did not discuss BS with their GP. | Check-in: No positive prompts if the user smoked that week. | Check-in: No positive prompts if the user did not visit a GP/audiologist or obtain a hearing aid. | Check-in: No positive prompts if the user did not exercise that week. | Check-in: No positive prompts if the user did not eat healthily that week. | Check-in: No positive prompts if the user had no drink-free days that week. | Check-in: No positive prompts if the user did not engage in social activities that week. | N/A |
| **15.Self Belief (Helping users to build self-efficiacy )** | Coach: Offer encouragement, highlight strengths, emphasize past successes, and remind users of their motivation for change. | *(Same as the 1st column)* | *(Same as the 1st column)* | *(Same as the 1st column)* | *(Same as the 1st column)* | *(Same as the 1st column)* | *(Same as the 1st column)* | *(Same as the 1st column)* | *(Same as the 1st column)* |
| **16. Covert learning (mental rehearsal, Vicarious consequences)** | Video: Feature success stories to help users observe positive behaviours and outcomes. | *(Same as the 1st column)* | *(Same as the 1st column)* | *(Same as the 1st column)* | *(Same as the 1st column)* | *(Same as the 1st column)* | *(Same as the 1st column)* | *(Same as the 1st column)* | *(Same as the 1st column)* |
| *BA: Behavioral Activation BS: Blood Sugar BP: Blood Pressure Note. The 16 Behaviour Change Techniques (BCTs) were identified with reference to the BCT Taxonomy v1 (Michie, S., Richardson, M., Johnston, M., Abraham, C., Francis, J., Hardeman, W., … Wood, C. E. (2013). The Behavior Change Technique Taxonomy (v1) of 93 hierarchically clustered techniques: Building an international consensus for the reporting of behavior change interventions. Annals of Behavioral Medicine, 46(1), 81–95).* | | | | | | | | | |
