# Supplementary material for: ENHANCE (Tailored Intervention for Brain Health and Cognitive Enrichment for Cognitive Health), a Coach-Supported Digital Intervention for Dementia Prevention in Underserved Older Adults: Co-Design and Usability Study
Source: J Med Internet Res. 2026 Jul 29;28:e91999. doi: 10.2196/91999 (PMC13469833; doi:10.2196/91999)
Supplement: Supplementary file 8 [file jmir_v28i1e91999_app2.docx]

| **GUIDED Item** | **Recommendation** | **Where addressed** | **Section/Page** |
| --- | --- | --- | --- |
| **1. Name** | Provide intervention name | ENHANCE (Empowering people to reduce dementia risk through a personalized digital prevention programme) | Title, P.1 |
| **2. Delivery** | Mode(s) of delivery | Blended: App-based and coach-supported | Abstract P.2-3  Background P.4-5 |
| **3. Problem** | Why intervention needed | Rising dementia rates, modifiable risk factors, health inequalities, Poor app engagement | Background P.4-5 |
| **4. Target group** | Intended recipients | cognitively healthy adults 50 years or above, including underserved populations | Background P. 4-5  Methods (Stakeholders & Recruitment) P.7 |
| **5. Stakeholders** | Who was involved | Interdisciplinary team of clinical academics, dementia risk–factor researchers, app developers, adults from underserved backgrounds (research participants), PPI contributors, and human–computer interaction experts. | Method (Stakeholders & Recruitment) P.7 |
| **6. Theory/evidence** | Theoretical foundation | Medical Research Council (MRC) framework for developing complex interventions; Double Diamond model; Person-Based Approach | Method P.5-6  Figure 1 |
| **7. Recipient involvement** | Target population participation | 199 individuals contributed to co-production activities: 162 research participants, 33 PPI contributors, and 4 human–computer interaction experts | Result (Participants) P.14-16  Table 1 |
| **8. Guiding principles** | Design principles | The 7 design principles: Integrate evidence-based BCTs in the app; Strength-based; Minimise cognitive and digital burden; Use of gamification features & familiar/classic games; Design for rapid change; Minimise external navigation; Promote autonomy and tailor to diverse capabilities. | Result (Guiding principles informing intervention design) P.18  Table 2 (Guiding principles) |
| **9. Development process** | Activities undertaken | Four-phase process: Discovery (evidence scan, usability testing), Define (principles, risk factor modules, game concepts, coaching roles), Design (iterative wireframe/prototype testing) and Deliver (one-week testing) | Figure 1  Result P.17-23 |
| **10. Changes during development** | Evolution through testing | Games: reduced difficulty, improved instructions, clearer feedback. Modules: simplified language, diverse imagery, customization features, additional content. App: resolved technical bugs and refined reward system | Result P.17-23 |
| **11. Final intervention** | Components, materials, procedures | Meadow-themed app; risk-factor modules (including videos, check in questions), 7 cognitive games, progress tracker, coaching sessions | Result- Final Prototype P. 22-23  Table 3 |
| **12. Uncertainties** | Unresolved issues | App refinements: accessibility settings, icon placement, reward system design. Future research: long-term engagement, real-world implementation barriers, scalability and deliverability of the coaching model | Discussion P. 24-29 |
