# Supplementary material for: ENHANCE (Tailored Intervention for Brain Health and Cognitive Enrichment for Cognitive Health), a Coach-Supported Digital Intervention for Dementia Prevention in Underserved Older Adults: Co-Design and Usability Study
Source: J Med Internet Res. 2026 Jul 29;28:e91999. doi: 10.2196/91999 (PMC13469833; doi:10.2196/91999)
Supplement: Supplementary file 9 [file jmir_v28i1e91999_app3.docx]

| **Example search strategy for hypertension** | |
| --- | --- |
| **Search component** | **Example terms** |
| **Risk factor component** | Hypertension OR “high blood pressure” |
| **Interventions** | intervention OR program* OR treatment OR management |
| **Population** | “older adult*” OR elderly OR ageing OR “middle age” |
| **Publication type** | (“Systematic review” OR “meta-analysis”) AND ("randomized controlled trial" OR "randomised controlled trial" OR RCT OR "clinical trial" OR "random allocation") |
