# Supplementary material for: ENHANCE (Tailored Intervention for Brain Health and Cognitive Enrichment for Cognitive Health), a Coach-Supported Digital Intervention for Dementia Prevention in Underserved Older Adults: Co-Design and Usability Study
Source: J Med Internet Res. 2026 Jul 29;28:e91999. doi: 10.2196/91999 (PMC13469833; doi:10.2196/91999)
Supplement: Supplementary file 10 [file jmir_v28i1e91999_app4.docx]

## **Detailed procedure and results of app selection for usability testing**

1. **Procedures of app selection**

To identify suitable apps for usability testing, we conducted a six-step process. This involves: (1) Searches for cognitive training and lifestyle apps  (2) eligibility screening using predefined inclusion and exclusion criteria; (3) information collection on app features and performance; (4) App quality and behavior change assessment using validated tools (MARS and ABACUS); and (5) ranking to select apps with the strongest evidence of  user engagement and scientific evidence and (6) Discussion.  Each step is described in detail below.

**1.1 App search:**

We conducted an app search between February and April 2023 to identify cognitive training and lifestyle apps for usability testing.

To identify cognitive training apps, we conducted a Google search (for the first 50 results) using the terms “brain training game” and “cognitive training game,” and supplemented a search of apps mentioned in published systematic reviews of cognitive training interventions for older adults.

For lifestyle apps, we began by identifying NHS-recommended apps. Using Google, we searched the terms “NHS mental health apps,” “NHS health apps,” and “NHS recommended apps”. As few NHS-endorsed apps were available for most of the targeted risk factors, we expanded our search to include commercial apps by using additional keywords such as “mobile apps,” “behaviors change,” “older adults,” and the names of specific risk factors. We also hand-searched systematic reviews to identify further relevant apps that meet our inclusion criteria.

We compiled all the app names in an Excel spreadsheet and removed the duplicates.

**1.2. App inclusion and exclusion criteria, and screening**

Following the app search, we screened all identified apps using pre-established inclusion criteria.

For **cognitive training apps**, we included those that were:

- Specifically designed for brain training through repeated practice; and
- Game-based.

For **lifestyle apps**, we included those that:

- Targeted improvement in at least one modifiable dementia risk factor addressed in ENHANCE (i.e., hypertension, diabetes, physical inactivity, obesity, depression, smoking, excessive alcohol use, social isolation, or hearing loss); and
- Included at least one behaviour change component, such as self-monitoring, goal setting, or habit formation.

Apps that only provided general health information without any active behavioral change component were excluded.

For both cognitive training and lifestyle apps, we included only English-language apps available for use on tablets or mobile devices. Apps designed for children or adolescents and those unavailable in the UK were excluded.

We assessed eligibility by reviewing app descriptions on official websites, the Apple App Store, and the Google Play Store. Apps were excluded if insufficient information was available to confirm their eligibility.

**1.3. Collecting information of the apps included:**

After app screening, we collected data on the characteristics of the apps included. The data we collected includes app name, version number, developer, subscription cost, affiliation with a professional medical/health association (Yes /No), available languages, country of origin, risk factor targeted , cognitive domains covered (for cognitive training apps only),  number of games (for cognitive training apps only), number of downloads from app market (Apple and Google), and average user ratings from the app market (Apple and Google). We collected this data from the official website and the Google and Apple app stores.

**1.4. Assessing app quality and behavior change techniques of apps:**

For all the apps included, two researchers independently assessed the quality and potential for behaviour change using the Mobile App Rating Scale (MARS) and App Behaviour Change Scale (ABACUS).

MARS is a validated scale that assesses app quality based on functionality, aesthetics, content, and engagement (Stoyanov et al., 2015). The scale consists of 23 items, each rated on a 5-point Likert scale, ranging from 1 (inadequate) to 5 (excellent). MARS generates a total score, along with four sub-scores representing functionality, aesthetics, content, and engagement. All scores range from 1 to 5, with higher scores indicating better app quality in the respective areas. We only used the total score of MARS when evaluating the apps.

ABACUS is a validated scale that evaluates the potential of an app to facilitate behavioural change (McKay et al., 2019) . It measures how well an app incorporates behaviour change techniques (BCTs) known to influence users' behaviours,. ABACUS consists of 21 questions and is organised into four key domains. 1. Knowledge and Information: Evaluates how effectively the app provides relevant information to raise awareness and emphasize the importance of the behavior. 2. Guidance and Support: Assesses whether the app offers personalized advice, prompts, or feedback to encourage the behavior. 3. Goal Setting and Tracking: whether the app helps users set specific behavior goals, monitor progress, and provides reinforcement to achieve these goals. 4. Social Support: whether the app enables users to connect with others for motivation and encouragement. Each question is rated with a Yes/No response. The ABACUS generates a total score ranging from 0 to 21, with higher scores indicating greater potential for facilitating behaviour change.

The two researchers downloaded all the eligible apps on a tablet, and independently interacted with each app for 10 minutes, and rated the eligible apps using MARS and ABACUS. They resolved any discrepancies through discussion or by averaging the scores. The MARS and ABACUS total score, along with the descriptive data from the previous steps, was combined in an excel spreadsheet.

**1.5. Identifying apps with the strongest evidence of user engagement and scientific research**

To identify apps with the strongest evidence of positive user engagement and scientific support, we applied a framework to quantify these two dimensions (Table 1).  Table 1 outlines the parameters used to assess each dimension.

| **Table 1.  Factors to consider for apps selection** | |
| --- | --- |
| **Factors to consider** | **Parameters** |
| 1. Level of user engagement * | - **App Quality**: This was assessed by two researchers using the Mobile application rating scale (MARS). The total score of MARS ranged from 1-5, with higher scores indicating better apps quality. - **Consumer ratings of the apps:** Average star rating form Apple App Store & Google Play store.  The rating ranges from 0-5, with higher scores indicating better customer’s rating. † - **Level of Popularity:** Monthly download rates of the app (Total no. of downloads/ Number of months that the app had been launched to the market) † |
| 2. Level of evidence   * | - **Evidence on efficacy:** A single item question from MARS was used to assess the scientific evidence on efficacy. ‘Has the app been trialed/tested in high quality randomized controlled trial before?’ This question is a 5-point Likert scale question, ranging from 1-5. A higher score indicates a higher level of scientific evidence on efficacy. We collected peer-reviewed papers for each app, and gave an evidence-based score to each app. - **Science on behavioral change:** The App behavioural change potential was assessed by App Behavioural Change Scale (ABACUS). The ABACAUS creates a total score, ranging from 0-21, with a higher score indicating a higher level of potential in changing behavior. |
| - * We employed a ranking aggregation method (Li et al., 2019; Lin, 2010) to standardize the scores across parameters in terms of "level of user experience" and "level of scientific evidence". Each parameter was ranked compared to other apps, and the average rank was calculated to guide our discussion. - † I collected the star ratings, number of downloads, and the number of months that the app had been launched to the market for the 14 apps in June 2023. All this information were collected in the Google Play Store and Apple App store focusing on the latest versions of the app. - # I collected peer-reviewed RCT research papers for each app by hand-searching the reference lists of all systematic reviews related to cognitive training apps. I also contacted the app company to identify these papers. As the MARS single-item question requires raters to judge whether the RCT is a high quality one or not, I used the Cochrane Collaboration’s Risk of Bias Tool (Higgins et al., 2011) to assess the RCT’s study quality of each app. | |

We applied a ranking aggregation method to standardise the scores for both user engagement and scientific evidence (Li et al., 2019; Lin, 2010). Each parameter (e.g., consumer rating, MARS score, download numbers) was ranked relative to the other included apps, and average ranks were calculated for user experience and scientific evidence for each app. This process produced two composite scores for each app—one reflecting user engagement and the other scientific evidence. Each app was then plotted on a two-dimensional graph, with user engagement on one axis and scientific evidence on the other, to visualise the relative positioning of all apps.

**1.6. Discussion of apps selection for further usability testing**

We used the graphs (shown below) as a starting point for discussion to identify apps with strong evidence of user experience and scientific support. The research team then reviewed each app’s suitability for older adults from underserved backgrounds, considering factors such as app concept, user interface design, and ease of navigation. Priority was given to apps demonstrating both positive user feedback and robust scientific evidence, while also being appropriate for older adults. The selected apps were subsequently used for usability testing.

**2.Results of app selection**

Below, we present the results of app selection for cognitive training apps and lifestyle apps (with accompanying graphs). Apps highlighted in orange on the graph represent the final selected apps.

**2.1. Cognitive Training app selection for user testing**

Of nine identified apps, six (BrainHQ, Cognifit, Lumosity, NeuroNation, Elevate, Peak) showed good user engagement and scientific support. Peak was excluded because its games were too challenging for users with lower educational attainment and Elevate was excluded due to its heavy reliance on language-based tasks. Four apps—Cognifit, BrainHQ, Lumosity, and NeuroNation—were therefore selected for user testing.

App Selection for cognitive training games

| User Engagement rank | Scientific Evidence Rank |
| --- | --- |
| 6.33333 | 3.5 |
| 2.33333 | 3 |
| 5.66667 | 2.5 |
| 6.33333 | 2.5 |
| 5.33333 | 4 |
| 3 | 4 |
| 2.33333 | 1.5 |
| 3.33333 | 2.5 |
| 3 | 1 |

| User Engagement rank | Scientific Evidence Rank |
| --- | --- |
| 4 | 0 |
| 4 | 10 |

| User Engagement rank | Scientific Evidence Rank |
| --- | --- |
| 0 | 2.5 |
| 10 | 2.5 |

**2.2. Lifestyle app Selection for user testing**

**Drinking:** Of the five apps identified (Step Away, Drinkaware, DrinkLess, AlcooQuizz, Drink Free Days), only Drink Free Days demonstrated strong scientific evidence and positive user feedback. It was also deemed suitable for older adults and hence selected for user testing.

**Smoking:** We identified two apps (Smoke Free, Quit Smoking), both of which demonstrated a good level of scientific evidence and positive user feedback. We selected Smoke Free over Quit Smoking, as it is the most recent and updated version of the same app.

**Depression:** Of the five apps identified (MoodMission, Calm, Mindfulness, and Headspace, Wysa), four demonstrated strong user engagement and scientific evidence. We selected MoodMission, which is based on behavioral activation, over the three mindfulness-based apps (Calm, Mindfulness, Headspace), as it focuses on simple, action-oriented goals achievable within a short timeframe—making it particularly suitable for the project’s target population.

**Physical Inactivity:** We identified six apps, of which four demonstrated a good level of user experience and scientific evidence (Couch to 5K, Zombies, Run!, MyFitnessPal, Active10). We selected Active 10 as it was considered the most suitable for older adults. We excluded Zombies, Run! and Couch to 5K, as both focus on running, which may be less achievable for the target population, and the zombie theme may not be engaging for older adults. We also excluded MyFitnessPal, as its primary focus is on calorie balance rather than physical activity.

**Obesity:** We identified three apps (Weight Loss Plan, MyFitnessPal, and Lose It!); however, all relied primarily on calorie-deficit approaches. Given evidence that weight loss in older adults may be associated with increased mortality risk and other adverse outcomes, we chose not to include any of these apps. Instead of user-testing obesity apps, we user-tested alternative materials (e.g., videos) for this risk factor.

**Hypertension:** We identified three apps at the time—OMRON, Medisafe, and Blood Pressure Monitor. We decided not to select any of these for usability testing, as all demonstrated limited scientific evidence and low levels of user engagement.

**Diabetes:** We identified three apps for diabetes (Glucose Buddy, Medisafe, Diabetes Diary), of which only Glucose Buddy showed strong scientific support and high user engagement. However, it was excluded because it is designed for individuals already managing diabetes through regular blood glucose monitoring and established self-management routines. In the ENHANCE study, the target population consists of individuals with untreated or unmanaged diabetes, who typically do not monitor their glucose, may lack diagnosis or medication, and require support focused on awareness, and treatment initiation rather than ongoing glucose tracking.

**Reference**

Higgins, J. P. T., Altman, D. G., Gøtzsche, P. C., Jüni, P., Moher, D., Oxman, A. D., Savović, J., Schulz, K. F., Weeks, L., & Sterne, J. A. C. (2011). The Cochrane Collaboration’s tool for assessing risk of bias in randomised trials. *BMJ*, *343*(7829), 889–893. https://doi.org/10.1136/bmj.d5928

Li, X., Wang, X., & Xiao, G. (2019). A comparative study of rank aggregation methods for partial and top ranked lists in genomic applications. *Briefings in Bioinformatics*, *20*(1), 178–189. https://doi.org/10.1093/bib/bbx101

Lin, S. (2010). Rank aggregation methods. *WIREs Computational Statistics*, *2*(5), 555–570. https://doi.org/https://doi.org/10.1002/wics.111

McKay, F. H., Slykerman, S., & Dunn, M. (2019). The App Behavior Change Scale: Creation of a Scale to Assess the Potential of Apps to Promote Behavior Change. *JMIR Mhealth Uhealth*, *7*(1), e11130. https://doi.org/10.2196/11130

Stoyanov, S. R., Hides, L., Kavanagh, D. J., Zelenko, O., Tjondronegoro, D., & Mani, M. (2015). Mobile App Rating Scale: A New Tool for Assessing the Quality of Health Mobile Apps. *JMIR MHealth UHealth*, *3*(1), e27. https://doi.org/10.2196/mhealth.3422
