# Supplementary material for: ENHANCE (Tailored Intervention for Brain Health and Cognitive Enrichment for Cognitive Health), a Coach-Supported Digital Intervention for Dementia Prevention in Underserved Older Adults: Co-Design and Usability Study
Source: J Med Internet Res. 2026 Jul 29;28:e91999. doi: 10.2196/91999 (PMC13469833; doi:10.2196/91999)
Supplement: Supplementary file 11 [file jmir_v28i1e91999_app5.docx]

**Example of think-Aloud Usability Testing Interview Guide for Selected Apps (Lumosity)**

| **Introduction (3-5 mins)** | - **Researcher introduces self.** - **Introduces Lumosity:** You will be using an app called ‘LUMOSITY’. which is a brain training app designed to provide a fun workout for your brain. The games may help you improve your thinking and memory. - **What to do:** Throughout the testing, I will ask you to play a few brain games. You can choose which games you will be playing. I’ll ask about your thoughts and impressions. - Please remember that there are no right or wrong answers. I am not evaluating your skills; but are interested in your experience as a user. - **Recording:** I will be audio recording this, so I don’t miss anything you say. I will also take notes. - **Any questions:** Before I proceed, do you have any questions about the study or the testing process? If not, we can start right away. | |
| --- | --- | --- |
| **Step 1: Participants browses through the game tab. They select and play the first brain game in Lumosity (5-10 mins)**   | - **Researcher shows participant the ‘Game page’.** - **Research asks users to browse through the page:** Would you like to browse through this page yourself for a while and see what is on it? - **Encourage think aloud:** When browsing through, you can share your thoughts out loud…. You can simply just say anything that come into your mind or observe, like or dislike, any questions that come to mind. - **Researcher asks user to select a game:** If I ask you to select a game to start with, which one will you select? - **Researcher observes the participant playing the game.** - **Researcher makes notes on pauses & hesitations, body language, feedback and any questions raised.­** | **Alternative script (Use this only when participants need more guidance):**  **Introducing the game:** ‘On this page, you will find five categories of games: Memory games, Attention games, Problem solving Games, Flexibility games and Speed games. Each category contains around 4-6 different games.  ***•* Asks user to scroll through and see the variety available.**  • **Researcher asks user to select a game:** If I ask you to select a game to start with, which one will you select? You can start playing the game |
| **Step 2: Researcher interviews the participant about the 1^st^ game if there are things which had not been covered (5-10 mins)** | - **Once finished, Thank the participant for playing that game.** - **Invite user to share your thoughts on the game just played.**   **Questions (if not covered):**   - What was the experience of playing with the game like? - What makes you choose this game? - Did you find any aspects particularly enjoyable or challenging? - I noticed that you _, can you tell me why? - What features do you think could be improved? - What, if anything, did you find frustrating? - How easy or difficult is it to play this game?   **•Thank you for sharing that. Your feedback is valuable to us.**  **•Any Questions:** Before we move on, ask if they have any other comments/ suggestions about the game. | |
| **Step 3: Participant selects the 2nd game and plays it (5-10 minutes) and researcher collects feedback** | - Thank you for playing the first game. - **Select 2^nd^ game:** Would you like to continue and choose a different game from a different category. - Researcher observes the participant playing the game.­ - Researcher makes notes on pauses & hesitations, body language, feedback and any questions raised.   [Repeat steps 2 and 3 until the total duration of 45 minutes is reached] | **Alternative script (Use this only when participants need more guidance):**     - **Select 2^nd^ game:** You can choose another game from a different game category than the one you chose in the first step. - Confirm with the participant the game they have just selected: You just selected [game category]. This time, you can choose [category that are available] - Once you've made your selection, you can start to play the game right away. |
| **Step 4. Re Obtaining overall feedback about Lumosity (10 mins)** | - **Asking for general feedback:** You had now gone through [number of games] games. Can I hear your overall comments about this app?   **Questions:**  -How do you find the overall experience of playing Lumosity?  - Did you find any aspects of the app particularly enjoyable or challenging?  -What are your thoughts on the design and layout? [if the participants find any difficulty in answering this, researcher can ask about the colour, font size and visuals of this app?]  -What are your thoughts on the language that this app used?  - Are there any aspects that can be improved? [Probing: How would you change this app make it easier to use/ more attractive to use?] | |
| **Conclusion (1-2 mins)** | - Thank user for their valuable participation. - “Your feedback has been very helpful to us. - Ask user if they have any final thoughts or suggestions about their experience with the Lumosity app? - If not, end session and give £20 voucher to participant. | |
