# Supplementary material for: ENHANCE (Tailored Intervention for Brain Health and Cognitive Enrichment for Cognitive Health), a Coach-Supported Digital Intervention for Dementia Prevention in Underserved Older Adults: Co-Design and Usability Study
Source: J Med Internet Res. 2026 Jul 29;28:e91999. doi: 10.2196/91999 (PMC13469833; doi:10.2196/91999)
Supplement: Supplementary file 12 [file jmir_v28i1e91999_app6.docx]

## Heuristics Evaluation workbook

**Instruction:**

**The ENHANCE app is designed to lower dementia risk for individuals aged 60-80, particularly focusing on those with lower education, digital skills, and socio-economic status. It offers 8 brain training games and additional interventions addressing lifestyle factors. Please take approximately 10-15 minutes in exploring the app and engaging with all cognitive training games. Afterwards, please utilize the provided workbook to document identified issues and offer recommendations.**

| **Heuristic evaluation of the overall app**    **This table is to understand the issues of the overall ENHANCE app.  Please put on your answers in the issues and recommendations boxes.** | | | | |
| --- | --- | --- | --- | --- |
|  | **Heuristics principles** | **Description** | Issues | Recommendations |
| 1. | Feedback and Visibility of app Status | - A well-designed app should update users on ongoing activities, offering timely feedback. For instance, display 'loading...' during page transitions and indicate 'message sent' when users send a message to the coach.      - Does the app use visual cues or indicators to inform users of ongoing processes or completed actions? - Is there a balance between keeping users informed and avoiding unnecessary interruptions? |  |  |
| 2 | Match Between System and the Real World | - The app design should use plain language, using words and concepts familiar to the user, and avoiding jargon. - Ensure that the app design adheres to real-world conventions, presenting information in a natural and logical sequence. For instance, arrange information from left to right, top to bottom, and employ control icons that resemble familiar concepts (e.g., using a Trash icon for deletion). |  |  |
| 3 | User control and freedom | The app needs clear ‘return’ or " exit" button to quickly leave the unwanted action without going through a long process.   - Does the design allow users to go back a step in the process?? - Are exit button easily discoverable in the app or in the game? | Pairs games- what cognitive domains does that train?  -The black thing there in the game (word is distracting |  |
| 4 | Consistency (internal consistency and external consistency) | The app icons, texts and users’ action, style and layout should be consistent across different pages of the app.  (Internal consistency). And the app should align with the habits/preference of older adults (external consistency).     - Does the app icons, texts, and user actions consistent across various pages? - Does the style and layout remain the same throughout the app? - Is the app's interface intuitive and familiar to older adults, aligning with their expectations? |  |  |
| 5. | Recognition rather than recall | Users shouldn't have to recall information across different parts of the app.     - Are important details such as labels, menu items, and start icons consistently visible or easy to find across different parts of the app? - Can users easily find crucial information without having to remember it from one section to another? |  |  |
| 6. | Aesthetic and Minimalist Design | Ensure the designs is appealing to older adults and free from unnecessary information and have an appropriate amount of information.       - Does the design incorporate larger fonts and clear, easy-to-read text for older users? - Do you think the graphics in the app is suitable for older users? - Are colour contrasts chosen thoughtfully to enhance visibility and readability for older users? - Have you identified and removed any elements in the design that might be confusing or distracting for older users? - Is the layout uncluttered, focusing on essential features without overwhelming users with too much information? |  |  |
| 7. | Help Users Recognize, Diagnose, and Recover from Errors | The error messages should use plain language and familiar error visuals (e.g. bold red to remind users with something) and provide an immediate solution to resolve the error.      -Are error messages written in plain language, avoiding technical jargon or complex terminology?  - Do error messages use familiar visual cues, such as bold red text, to immediately catch the user's attention?  - Do error messages not only identify the problem but also provide clear and solutions for users? | (If no errors occurred during testing, please put N/A.) | (If no errors occurred during testing, please put N/A.) |
| 8. | Help and Documentation | A well-designed app should be intuitive enough so that older users can navigate and complete the task without requiring many explanations. But sometimes written instructions are needed for users to complete their tasks or actions.     - Does the ENHANCE app consist of a help button or FAQ (frequently asked questions) or guides? - Does these help available right away when the users need it and easy to find? |  |  |
| 9. | Error Prevention | - A well-designed app should try to avoid situations where mistakes are likely to occur (e.g., use a calendar widget to prevent users from entering the wrong date, employ a top-down checklist rather than asking participants to input the data, Not requiring older users to login repeatedly). Does ENHANCE have features in its design and interface to prevent potential mistakes made by users (e.g. entering unintended answer/ accidental click)?"      - If there is a chance a user might make a mistake, especially if that information is important, provide them with an opportunity to confirm their actions before finalizing (e.g., through a confirmation pop-up window). Does the ENHANCE app give users a chance to confirm important actions, and are there pop-up messages to help prevent accidental mistakes? |  |  |
| 10. | Flexibility and Efficiency of Use | The app should cater to the needs every targeted user, including those with different education levels, digital skills, ethnic backgrounds, and special needs like dyslexia or colour blindness.     - Does the enhance app contains customisation feature so that it can fit to everyone preference? (e.g. Dyslexia mode, colour blinded game mode) |  |  |
| **Heuristics principles for games on older adults**    **This table is to understand the issues of the cognitive training games in ENHANCE.  Please put on your answers in the issues and recommendations boxes.** | | | | |
| 1 | Clarity of Rules and Objectives | The game should provide clear rules, present the main objectives at the beginning.    -Are the main objectives of the game clearly communicated to players at the beginning?  -Are the rules of the game presented in a clear and concise manner? |  |  |
| 2 | Effective Tutorial | - The game should provide an interesting, engaging, and absorbing tutorial. - The tutorial should simulate gameplay, facilitating easy understanding. |  |  |
| 3 | Inclusive Visual and Audio Design | - Visual and audio effects should be utilized to arouse interest and interaction of older adults. |  |  |
| 4 | Enjoyable Gameplay | - The game should be fun and enjoyable, avoiding boring tasks. - Boredom can be minimized by varying activities and increasing difficulties during play. |  |  |
| 5 | Easy to learn. | - The game should be easy to learn and should not be difficult to understand how to play. The players must feel in control. - The game must teach the basic skills needed at the beginning (basic levels), to be used later by the players. |  |  |
| 6 | Rewards system | - The game should provide rewards which should be meaningful to older adults, encouraging them to play more. - The older player should not be penalized for the same error. - Player should not lose any earned rewards. |  |  |
| 7 | Optimal level of difficulty of the games and slow progression | -Challenges should be consistent with the ability of older people, making the player want to play more, rather than giving up.  - The level of difficulty should increase gradually at a pace that does not generate frustration. |  |  |
| 8 | Duration of the game | - The duration of gameplay should strike a balance, avoiding excessive length that may lead to boredom, while ensuring it is not too brief, leaving players feeling unsatisfied. |  |  |
| 9 | Immediate Feedback | - The game should clear, providing immediate feedback. This ensures players understand why they earn or not earn a game point. |  |  |
| 10 | Progress Visualisation | - The player must be able to visualize their progress in the game and also to compare the results. |  |  |

Reference:

<https://media.nngroup.com/media/articles/attachments/Heuristic_Evaluation_Workbook_1_Fillable.pdf>

Machado, M. da C., Ferreira, R. L. R., & Ishitani, L. (2018). Heuristics and Recommendations for the Design of Mobile Serious Games for Older Adults. *International Journal of Computer Games Technology*, *2018*, 1–15. <https://doi.org/10.1155/2018/6757151>
