# Supplementary material for: ENHANCE (Tailored Intervention for Brain Health and Cognitive Enrichment for Cognitive Health), a Coach-Supported Digital Intervention for Dementia Prevention in Underserved Older Adults: Co-Design and Usability Study
Source: J Med Internet Res. 2026 Jul 29;28:e91999. doi: 10.2196/91999 (PMC13469833; doi:10.2196/91999)
Supplement: Supplementary file 13 [file jmir_v28i1e91999_app7.docx]

| **Criterion domain** | **Operational definition / exit criterion** | **Assessment method** |
| --- | --- | --- |
| **Technical quality** | **No critical or high-priority defects or bugs identified during final test cycles** | **Internal technical testing and bug-tracking logs *(Described in*** ***‘Technical and expert evaluation’******)*** |
| **Usability** | **Participants completing at least once three cognitive games, one check-in activity, and one video viewing (core tasks) during the one-week testing period** | **One-week user testing *(Described in ‘******Extended one-week use testing******’)*** |
| **Behavioural fidelity** | **Alignment with intended behaviour change techniques (BCTs) across all key modules** | **BCT mapping table reviewed by the research team (Described in Section** **‘Intervention-level behaviour changes technique alignment’****)** |
| **Functional stability** | **Stable performance across study devices, with no crashes or data loss** | **Device-level testing and backend monitoring** |
| **Safety and acceptability** | **No risks, distress, or adverse reactions identified** | **Researcher observation, participant feedback during one-week testing** |

Exit criteria for development of app and assessment methods
