# Supplementary material for: ENHANCE (Tailored Intervention for Brain Health and Cognitive Enrichment for Cognitive Health), a Coach-Supported Digital Intervention for Dementia Prevention in Underserved Older Adults: Co-Design and Usability Study
Source: J Med Internet Res. 2026 Jul 29;28:e91999. doi: 10.2196/91999 (PMC13469833; doi:10.2196/91999)
Supplement: Supplementary file 15 [file jmir_v28i1e91999_app9.docx]

| **Primary intervention strategy for each risk factor module in ENHANCE** | | |
| --- | --- | --- |
| **Risk factor** | **Primary Approach** | **Rationale for the Selection** |
| Depression | Behavioural Activation (BA) | BA was selected for its large effect in reducing depression among older adults [1], its focus on simple, achievable goals, and suitability for non-specialist delivery and lower-literacy users. Its action-oriented approach also lends itself well to app-based delivery. Other effective interventions — including CBT, cognitive therapy, life review therapy, and mindfulness — were not considered, as they require specialist facilitation and complex cognitive or reflective processing, making them less suitable for this context.[2–4] |
| Social isolation | Social engagement to reduce isolation | A social engagement approach — promoting direct interactions with family, friends, and community groups — was selected for its effectiveness in reducing social isolation, [5]. feasibility within six weeks, and suitability for non-specialist, app-based delivery. Animal-assisted therapy, though also effective, was excluded as it is not feasible to deliver digitally [6,7] |
| Hearing loss | Hearing health awareness and early management | A hearing health awareness approach — focused on understanding the importance of early hearing loss management (including hearing aid use) — was selected given evidence linking hearing aids to reduced dementia risk [8]. Awareness-raising was prioritised over intervention as a more achievable goal within six weeks, particularly given UK hearing aid waiting times. Structured communication training, though effective [9], was excluded as it requires specialist input and is less suited to app-based delivery. |
| Hypertension | BP self-monitoring and care-seeking approach | We adopted a focused self-management approach centred on regular home blood-pressure monitoring and appropriate action on elevated readings (e.g., consulting a GP). This was selected for its short-term effectiveness, feasibility within a 6-week app-based intervention, and alignment with UK clinical guidelines, which emphasise home BP monitoring for diagnosis and treatment decisions. |
| Diabetes | Diabetes awareness and early self-management | A brief intervention focused on early-stage diabetes self-management was adopted, covering lifestyle skills (diet, physical activity) and encouraging healthcare consultation for those not yet seeking medical advice. This aligns with clinical guidelines recommending lifestyle modification as a first-line strategy, is achievable within a short intervention period [10], and is well-suited to app-based delivery. Pharmacotherapy was excluded [11] as it requires clinical oversight and is not suitable for digital implementation. |
| Smoking | Smoking cessation through brief behavioural intervention | A brief behavioural intervention was adopted, helping participants set a quit date and providing weekly guidance through behaviour change techniques (e.g., personalised feedback, self-monitoring). This approach is effective for smoking cessation in older adults [12,13], aligns with NHS guidance on quit-date setting and structured planning, and is more feasible for coach-supported app-based delivery than alternatives such as pharmacotherapy or psychotherapy [13]. |
| Alcohol | Alcohol reduction through controlled drinking | A brief behavioural intervention was adopted, encouraging alcohol-free days and providing weekly guidance through behaviour change techniques (e.g., personalised feedback, self-monitoring). Controlled drinking was prioritised over abstinence as a more realistic and effective approach to reducing consumption within six weeks [14,15], and aligns with NHS guidance recommending at least two alcohol-free days per week. |
| Obesity | Weight management through combined diet and exercise | A combined diet and exercise approach was selected, as integrating both components improves weight loss effectiveness in older adults [16]. Healthy eating recipes and exercise videos were tailored to accommodate varying dietary needs and physical capabilities. |
| Physical inactivity | Physical activity promotion using behaviour change techniques | A brief behavioural intervention was adopted, integrating behaviour change techniques (self-monitoring, feedback, goal setting), education, and home-based exercise video demonstrations. This approach is effective in increasing physical activity among older adults [17–19] and is well-suited to app-based delivery within a six-week period. |

**Reference**:

1.	Orgeta V, Brede J, Livingston G. Behavioural activation for depression in older people: systematic review and meta-analysis. British Journal of Psychiatry 2018/01/02. Cambridge University Press; 2017;211(5):274–279. doi: 10.1192/bjp.bp.117.205021

2.	Lin J, Zhao R, Li H, Lei Y, Cuijpers P. Looking back on life: An updated meta-analysis of the effect of life review therapy and reminiscence on late-life depression. J Affect Disord 2024;347:163–174. doi: https://doi.org/10.1016/j.jad.2023.11.050

3.	Ye J, Pan Y, Wu C, Hu Z, Wu S, Wang W, Guo J, Xiao A. Effects of Mindfulness-Based Cognitive Therapy on Depression and Anxiety in Late Life: A Meta-Analysis. Alpha Psychiatry 2024/05/27. 2024;25(1):1–8. PMID:38799491

4.	Morgado B, Silva C, Agostinho I, Brás F, Amaro P, Lusquinhos L, Silva MR, Fonseca C, Albacar-Riobóo N, Guedes de Pinho L. Psychotherapeutic Interventions for Depressive Symptoms in Community-Dwelling Older Adults: A Systematic Review with Meta-Analysis. Healthcare 2024;12(24):2551. PMID:doi:10.3390/healthcare12242551

5.	Gardiner C, Geldenhuys G, Gott M. Interventions to reduce social isolation and loneliness among older people: an integrative review. Health Soc Care Community John Wiley & Sons, Ltd; 2018;26(2):147–157. doi: https://doi.org/10.1111/hsc.12367

6.	Hoang P, King JA, Moore S, Moore K, Reich K, Sidhu H, Tan CV, Whaley C, McMillan J. Interventions Associated With Reduced Loneliness and Social Isolation in Older Adults: A Systematic Review and Meta-analysis. JAMA Netw Open 2022;5(10):e2236676–e2236676. doi: 10.1001/jamanetworkopen.2022.36676

7.	Dickens AP, Richards SH, Greaves CJ, Campbell JL. Interventions targeting social isolation in older people: a systematic review. BMC Public Health 2011;11(1):647. doi: 10.1186/1471-2458-11-647

8.	Yeo BSY, Song HJJMD, Toh EMS, Ng LS, Ho CSH, Ho R, Merchant RA, Tan BKJ, Loh WS. Association of Hearing Aids and Cochlear Implants With Cognitive Decline and Dementia: A Systematic Review and Meta-analysis. JAMA Neurol 2023;80(2):134–141. doi: 10.1001/jamaneurol.2022.4427

9.	Laplante-Lévesque A, Hickson L, Worrall L. Rehabilitation of Older Adults With Hearing Impairment: A Critical Review. J Aging Health 2010;22(2):143–153. PMID:20056812

10.	Alliston P, Jovkovic M, Khalid S, Fitzpatrick-Lewis D, Ali MU, Sherifali D. The effects of diabetes self-management programs on clinical and patient reported outcomes in older adults: a systematic review and meta-analysis. Frontiers in Clinical Diabetes and Healthcare 2024;5. doi: 10.3389/fcdhc.2024.1348104

11.	Pan SY, Su EL, Huang CJ, Chuang SY, Chiang CE, Chen CH, Cheng HM. Evaluation of glucose-lowering medications in older people: a comprehensive systematic review and network meta-analysis of randomized controlled trials. Age Ageing 2024/08/13. 2024;53(8). PMID:39137064

12.	Smith P, Poole R, Mann M, Nelson A, Moore G, Brain K. Systematic review of behavioural smoking cessation interventions for older smokers from deprived backgrounds. BMJ Open 2019;9(11):e032727. doi: 10.1136/bmjopen-2019-032727

13.	Chen D, Wu LT. Smoking cessation interventions for adults aged 50 or older: A systematic review and meta-analysis. Drug Alcohol Depend Elsevier; 2015;154:14–24. doi: 10.1016/j.drugalcdep.2015.06.004

14.	Kelly S, Olanrewaju O, Cowan A, Brayne C, Lafortune L. Alcohol in older people: systematic reviews of interventions and context. The Lancet Elsevier; 2016;388:S64. doi: 10.1016/S0140-6736(16)32300-5

15.	Boumans J, van de Mheen D, Crutzen R, Dupont H, Bovens R, Rozema A. Understanding How and Why Alcohol Interventions Prevent and Reduce Problematic Alcohol Consumption among Older Adults: A Systematic Review. Int J Environ Res Public Health. 2022. doi: 10.3390/ijerph19063188

16.	Batsis JA, Gill LE, Masutani RK, Adachi-Mejia AM, Blunt HB, Bagley PJ, Lopez-Jimenez F, Bartels SJ. Weight Loss Interventions in Older Adults with Obesity: A Systematic Review of Randomized Controlled Trials Since 2005. J Am Geriatr Soc John Wiley & Sons, Ltd; 2017;65(2):257–268. doi: https://doi.org/10.1111/jgs.14514

17.	van der Bij AK, Laurant MGH, Wensing M. Effectiveness of physical activity interventions for older adults: a review1 1The full text of this article is available via AJPM Online at www.elsevier.com/locate/ajpmonline. Am J Prev Med 2002;22(2):120–133. doi: https://doi.org/10.1016/S0749-3797(01)00413-5

18.	Taylor J, Walsh S, Kwok W, Pinheiro MB, de Oliveira JS, Hassett L, Bauman A, Bull F, Tiedemann A, Sherrington C. A scoping review of physical activity interventions for older adults. International Journal of Behavioral Nutrition and Physical Activity 2021;18(1):82. doi: 10.1186/s12966-021-01140-9

19.	Senkowski V, Gannon C, Branscum P. Behavior Change Techniques Used in Theory of Planned Behavior Physical Activity Interventions Among Older Adults: A Systematic Review. J Aging Phys Act 2019;27(5):746–754. doi: 10.1123/japa.2018-0103
