# Supplementary material for: ENHANCE (Tailored Intervention for Brain Health and Cognitive Enrichment for Cognitive Health), a Coach-Supported Digital Intervention for Dementia Prevention in Underserved Older Adults: Co-Design and Usability Study
Source: J Med Internet Res. 2026 Jul 29;28:e91999. doi: 10.2196/91999 (PMC13469833; doi:10.2196/91999)
Supplement: Supplementary file 18 [file jmir_v28i1e91999_app12.docx]

## Example of the initial hypertension workflow chart
